# Impact of heated tobacco products on biomarkers of potential harm and adverse events: a systematic review and meta-analysis

**DOI:** 10.1136/tc-2024-059000

**Published:** 2025-04-29

**Authors:** Sophie Braznell, Sarah Dance, Jamie Hartmann-Boyce, Anna Gilmore

**Affiliations:** 1 Tobacco Control Research Group, Department for Health, University of Bath, Bath, UK; 2 Department of Psychology, University of Bath, Bath, UK; 3 Department of Health Promotion and Policy, University of Massachusetts Amherst, Amherst, Massachusetts, USA; 4 Nuffield Department of Primary Care Health Sciences, University of Oxford, Oxford, UK; 5 Centre for 21st Century Public Health, Department for Health, University of Bath, Bath, UK

**Keywords:** Tobacco industry, Non-cigarette tobacco products, Harm Reduction

## Abstract

**Objective:**

To systematically review available data on the effects of heated tobacco products (HTPs) on biomarkers of potential harm (BoPH) and adverse events, including comparison to cigarettes, e-cigarettes and smoking abstinence.

**Data sources:**

Web of Science, Scopus, MedRxiv, ClinicalTrials.gov, ICTRP database and HTP manufacturer libraries were searched from January 2010 to December 2024.

**Study selection:**

Included studies were interventional clinical trials of any design that measured BoPH or adverse events in adults assigned a marketed HTP and another assigned either cigarettes, e-cigarettes or smoking abstinence.

**Data extraction:**

Two reviewers independently extracted data into a predesigned form and assessed risk of bias using Cochrane’s Risk of Bias tool version 1.

**Data synthesis:**

BoPH data were synthesised using effect direction plots. A random-effects model was used to calculate pooled risk ratios for adverse event data. 40 studies (10 independent, 29 industry-affiliated and 1 of unclear affiliation) were included. Only nine studies lasted longer than 5 days. 19 involved using the intervention just once. Risk of bias was high for 32 studies and unclear for 8. Data on 143 BoPH indicated short-term HTP use had mixed effects compared with cigarettes, e-cigarettes and smoking abstinence. The rate of adverse event reporting was not significantly different between HTP and any comparator group.

**Conclusions:**

Despite a growing evidence base, significant limitations hinder interpretation of the data, which do not yet provide clear indication of harm or benefit, even compared with cigarettes. Longer, better quality studies independent of tobacco industry funding are needed to determine the health impacts of HTPs.

## Introduction

 Heated tobacco products (HTPs) are designed to heat processed tobacco, instead of burning it like cigarettes, to produce an aerosol containing nicotine and other chemicals.^1^ Like electronic cigarettes (e-cigarettes), HTPs use an electronic component, but unlike e-cigarettes, HTPs contain tobacco leaf. The use of HTPs is increasing globally.^2^ This is partly attributable to the tobacco industry’s marketing, including claims that HTPs are ‘smoke-free’, ‘reduced-risk’ and ‘alternatives’ to cigarettes.^3^,^5^ Whether HTPs are ‘smoke-free’ or ‘alternatives’ to cigarettes has been contested by independent research.^6 7^ However, the health risks of HTPs and whether these risks are reduced compared with cigarettes remains unclear.^8^,^10^

We previously critically appraised the conduct and reporting of interventional clinical trials on HTPs.^11^ We found they were limited in quantity and quality. Most were funded by the tobacco industry, which has a history of scientific misconduct and hiding the harms of its products.^12^ In the absence of alternatives, data from these trials is used by policy-makers and regulators to inform life-changing decisions. For example, many of the existing trials were used to attain a marketing order for long-standing HTP leader, IQOS, from the US Food and Drug Administration (FDA), which is currently up for renewal.^10^ The uncertainties around the evidence to date exacerbate the challenges of regulators facing rising use, relentless harm reduction marketing and pressure from the tobacco industry to ease regulations for HTPs. Thus, despite concerns over quality and bias, synthesising the available data in this evolving area remains essential to provide clarity on the current state of evidence.

To our knowledge, biomarkers of potential harm (BoPH) and adverse event data from HTP clinical trials have yet to be systematically synthesised. In the absence of long-term epidemiological data, such indicators of clinical risk are particularly important to estimate the potential health impacts of HTPs. Further, previous reviews have often only included peer-reviewed literature,^913^,^16^ despite grey literature containing a significant portion of the available data.^11^

Building on our previous review, we aimed to present, as comprehensively as possible, the available data on the effects of HTPs on BoPH and adverse events. To achieve this, we sought to:

1. Identify available BoPH and adverse event data from clinical trials examining HTP use.
2. Examine the effect of HTP use on BoPH and adverse events.
3. Compare the effects of HTP use to those of cigarettes, electronic cigarettes (e-cigarettes) and smoking abstinence on BoPH and adverse events.

## Methods

This review builds on our previous review.^11^ We followed PRISMA (Preferred Reporting Items for Systematic Reviews and Meta-Analyses) guidelines^17^ and a protocol was registered on PROSPERO (CRD42021286807, https://www.crd.york.ac.uk/prospero/display_record.php?ID=CRD42021286807).

### Search strategy

We used the same search strategy as in the previous review (https://www.crd.york.ac.uk/PROSPEROFILES/240676_STRATEGY_20210429.pdf). In brief, Web of Science (Core Collection and MEDLINE), Scopus, MedRxiv, ClinicalTrials.gov, the International Clinical Trials Registry Platform (ICTRP) and transnational HTP manufacturers’ (Philip Morris International, PMI; British American Tobacco, BAT; Japan Tobacco International, JTI; Imperial Brands, IB) publication databases were searched using HTP and clinical trial terminology. Searches were restricted to literature published after 1 January 2010 to exclude studies on HTPs not available on the market during the past decade. The most recent searches were run on 5 December 2024.

### Study selection

Literature was managed in Covidence. Following removal of duplicates, eligibility screening was piloted by two reviewers on 20% of the literature. Titles and abstracts from the updated searches were screened independently by two reviewers (SD and SB). The same reviewers then independently assessed the full-text articles against the eligibility criteria. Disagreements were resolved via discussion. Cohen’s kappa (k) was calculated to measure inter-rater agreement.

Eligibility criteria:

- Design: interventional clinical trials (in which human participants are prospectively assigned an intervention to evaluate its effects on health-related outcomes)^18^ of any design and setting. Studies did not need to be peer-reviewed.
- Population: adult cigarette smokers and non-smokers (as defined by study authors).
- Intervention: HTPs marketed within the last decade.
- Comparison: combustible cigarettes, e-cigarettes or smoking abstinence (as defined by study authors).
- Outcome: BoPH or adverse events.

BoPH comprise any outcome that measures an effect due to a potentially harmful exposure, such as clinical endpoints, surrogate markers and biomarkers of risk or effect.^19^ Adverse event data were differentiated into serious adverse events and all other adverse events, as defined by study authors.

### Data extraction

Two reviewers (SD and SB) independently extracted study characteristics (trial registration ID, sponsor, affiliation, design, intervention groups), BoPH and adverse event data into a predesigned and piloted form in Covidence. BoPH data extracted were units, time point and analysis population for each biomarker, and mean, variance (SD or CIs) and the number of participants at baseline and last follow-up for HTP, cigarette, e-cigarette and smoking abstinence groups. Where baseline and last follow-up data were not reported, change from baseline and between-group differences were extracted as available.

The number of adverse and serious adverse events, the number of participants reporting these events and any information on the types of adverse events reported for each group were extracted.

Two reviewers (SB and SD) independently coded whether trials were associated with the tobacco industry using the coding scheme from the previous review (online supplemental appendix 1).

### Risk of bias assessment

Risk of bias was assessed using the Cochrane Risk of Bias tool version 1^20^; the preferred version of the Cochrane Tobacco Addiction Review Group. Following guidance in the Cochrane Handbook for Systematic Reviews of Interventions^20^ and from the Cochrane Tobacco Addiction Review Group,^21^ we assessed: random sequence generation; allocation concealment; blinding of participants and personnel; blinding of outcome assessment; incomplete outcome data; selective outcome reporting and other sources of bias. Recognising the difficulties in blinding studies involving visually distinct products, blinding of outcome assessment was rated low for unblinded trials in which the outcomes of interest for our review were objectively measured (eg, biochemical measures) and high in cases of self-report. Blinding of participants and personnel was rated low for unblinded trials in which the control(s) was an active intervention (eg, e-cigarettes or nicotine replacement therapy) of similar intensity (ie, all arms received the same co-interventions and/or support). Random sequence generation and allocation concealment domains were rated high for all non-randomised trials. Overall risk of bias was rated ‘low’ when there was low risk of bias in all domains, ‘unclear’ when there was unclear risk of bias in ≥1 domains, or ‘high’ when there was high risk of bias in ≥1 domains.^20^

The assessment was piloted in our previous review.^11^ Two reviewers (SD and SB) independently performed assessments. Risk of bias plots were generated using RobVis.^22^

### Data synthesis

Due to the quantity of different biomarkers measured, a paucity of studies measuring the same biomarkers, and heterogeneity of the data, we could not perform meta-analyses of BoPH data. Instead, we used effect direction plots. We identified the direction of effect for each biomarker between HTP and comparator arms at last follow-up. Although not our primary objective, we also established the direction of effect between baseline and last follow-up within just the HTP arms.

Statistical significance or magnitude of effect was not taken into consideration, as recommended by Cochrane.^23^ We determined whether the direction represented a beneficial or harmful effect based on information provided in included studies, clinical literature and effects caused by smoking or cessation. The direction indicative of harm for each biomarker is detailed in online supplemental table 1), alongside supporting literature. We determined the direction of effect on an intention-to-treat basis where possible. It was not possible to determine whether a change in reversibility in forced expiratory volume in 1 s, weight and waist circumference constitutes a beneficial or harmful effect without examining individual-level data. Therefore, these outcomes were excluded from analyses.

We conducted meta-analyses to investigate the relative risk of adverse events and serious adverse events following HTP use and cigarette use, e-cigarette use or smoking abstinence. Risk ratios (RRs) and 95% CIs were calculated for each trial using the number of participants that reported adverse events and combined using a Mantel-Haenszel random‐effects model in RevMan V.5.4.1. To avoid unit-of-analysis error, cross-over studies were excluded from this analysis. For studies with more than two intervention arms, data were combined across arms of the same intervention type (ie, HTPs, cigarettes or e-cigarettes). Data not included in the meta-analyses were synthesised narratively.

Under confined conditions (ie, controlled environments, like residential clinics), participants cannot deviate from the assigned intervention. Thus, the data from such studies represent exclusive use. Under ambulatory conditions (ie, uncontrolled environments, like participants’ homes) participants can deviate, for example, reverting to smoking. Thus, the data from ambulatory trials better emulate real-world use. Confined and ambulatory studies are discussed separately and subgrouped in meta-analyses.

## Results

### Included studies

Overall, searches identified 1634 records (online supplemental figure 1). Of these, 100 records relating to 40 studies met the inclusion criteria. There was good and very good^24^ inter-rater agreement for screening (k=0.69) and eligibility assessment (k=0.88), respectively. We found three additional trial registrations via the ID number referenced in studies already included, meaning a total of 103 records were included.

10 studies had no known tobacco industry affiliation (ie, independent), 29 were industry-affiliated and 1 affiliation was unclear for one study in which an HTP manufacturer provided the devices but, according to study authors, had no other involvement (table 1). 38 studies included at least one cigarette arm. Only six included at least one e-cigarette arm and nine included at least one smoking abstinence arm. Participants assigned HTPs in all but one study were current smokers at the time of enrolment and were exposed via inhalation (ie, used the HTP as designed). Participants in a novel clinical study (Dalrymple, 2022) were non-smokers and rather than use the interventions, their skin was exposed to product emissions. Given these differences, we discuss this study separately. Of the remaining 39 trials, 31 were conducted in confined settings, three in ambulatory, and five had a short confinement period immediately followed by an ambulatory period. As per our methods, we extracted last follow-up data, which in the latter five meant the end of the ambulatory period and not from the initial confinement period. Most trials had durations of 5 days or less, with participants in 19 trials only using the intervention once (single use, eg, one cigarette or heat stick) or for similarly short durations (eg, 14 puffs or 5–7 min). Only nine trials were longer, with the longest being 12 months. One trial included a single-use period followed by 90 min of use. Data were extracted from both periods as the mode of exposure differed (ie, restricted then ad libitum) and there was a 30 min break in-between.

**Table 1:** Overview of included studies

	Study	Sponsor	Design	Setting	Exposure duration	Exposure mode	Intervention arms	Risk of bias
**Industry-affiliated**	ISRCTN13439529* 57,59	BAT	Crossover RCT	Confined	Single use	Inhaled; ad libitum	HTPs (Glo1.0; Glo1.1), CC (own brand), NRT (Nicorette inhaler)	High
	ISRCTN14301360/UMIN000024988* 60,63	BAT	Parallel RCT	Confined	5 days	Inhaled; ad libitum	HTPs (Glo1.0 regular; Glo1.0 menthol; IQOS), CCs (Lucky Strike regular; Lucky Strike menthol), SA (all nicotine)	High
	ISRCTN80651909*† ^45 64^	BAT	Parallel RCT	Confined	5 days	Inhaled; ad libitum	HTPs (Glo; unknown), CC (Lucky Strike regular), EC (IS1.0(TT) prototype), SA (all nicotine)	High
	ISRCTN81075760*† 3365,71	BAT	Parallel RCT	Ambulatory	12 months	Inhaled; ad libitum	HTPs (Glo1.1; THD2.4T20), CC (own brand), SA (smoking)	High
	Dalrymple, 2022 ^25^	BAT	Novel skin stain study	Confined	32 puffs	Skin stained; restricted	HTP (Glo1.0), CC (commercial cigarette ‘N491’), EC (Vype ePen 3)	Unclear
	NCT05114863*† ^72 73^	RAI Services (BAT)	Crossover RCT	Confined	HTP: 3–4 min CC: 5 min NRT: 30 min	Inhaled; ad libitum	Regular HTPs (NeoSmooth Standard; NeoSmooth Boost; NeoCLICK uncrushed), Menthol HTPs (NeoFresh Standard; NeoFresh Boost; NeoSmooth Boost; NeoCLICK), NRT (Nicorette gum), CCs (own brand regular; menthol)	High
	NCT06093659*^74 75^	Imperial Brands	Crossover RCT	Confined	5 days	Inhaled; ad libitum	HTPs (PULZE 2.0 Balanced Blue; Rich Bronze), Heated herbal products (PULZE 2.0 Forest Berry; Summer Watermelon), CC (own brand)	High
	NCT05459857* ^76 77^	Imperial Brands	Crossover RCT	Confined	5 days	Inhaled; ad libitum	HTPs (PULZE intense; regular; menthol), CC (own brand)	High
	UMIN000017297* ^78 79^	JTI	Crossover RCT	Confined	10 puffs	Inhaled; restricted	HTP (novel tobacco vapour product), CC (unknown)	High
	UMIN000025777* 80,82	JTI	Parallel RCT	Confined	5 days	Inhaled; ad libitum	HTP (novel tobacco vapour product), CC (own brand), SA (smoking)	High
	UMIN000041539* ^83 84^	JTI	Parallel RCT	Confined	5 days	Inhaled; ad libitum	HTPs (Ploom TECH+; Ploom S; IQOS; Glo), CC (own brand), SA (smoking)	High
	UMIN000045304*^85 86^	JTI	Parallel RCT	Confined	5 days	Inhaled; ad libitum	HTPs (PLOOM X regular; menthol; IQOS 3 DUO), CC (own brand), SA (smoking)	High
	Yuki, 2023† ^87^	JTI	Parallel RCT	Confined (6 days) Ambulatory (54 days)	60 days	Inhaled; ad libitum	HTPs (IT2.0b regular; menthol green; menthol purple), ECs (eDNC1.0a tobacco; cherry; menthol), CC (own brand), SA (tobacco)	High
	NCT02641587*† ^34 88^	PMI	Parallel RCT	Confined (5 days) Ambulatory (85 days)	90 days	Inhaled; ad libitum	HTP (CHTP1.2), CC (own brand)	High
	NCT01959607* 89,92	PMI	Crossover RCT	Confined	14 puffs/6 min	Inhaled; restricted	HTP (IQOS), CC (own brand), NRT (Nicorette gum)	High
	NCT02503254* 93,96	PMI	Parallel RCT	Confined	5 days	Inhaled; ad libitum	HTP (CHTP1.0), CC (own brand)	High
	NCT01967719* 97,99	PMI	Crossover RCT	Confined	Single use	Inhaled; ad libitum	HTP (IQOS2.2 menthol), CC (own brand menthol), NRT (Nicotrol nasal spray)	High
	NCT01989156*† 35100,102	PMI	Parallel RCT	Confined (5 days) Ambulatory (86 days)	91 days	Inhaled; ad libitum	HTP (IQOS2.2 menthol), CC (own brand menthol), SA (smoking)	High
	NCT01970982*† 46103,105	PMI	Parallel RCT	Confined	5 days	Inhaled; ad libitum	HTP (IQOS2.2), CC (own brand), SA (all nicotine)	High
	NCT01959932*† 106,110	PMI	Parallel RCT	Confined	5 days	Inhaled; ad libitum	HTP (IQOS2.2), CC (own brand), SA (all nicotine)	High
	NCT01780714*† ^47 111 112^	PMI	Parallel RCT	Confined	5 days	Inhaled; ad libitum	HTP (IQOS2.1), CC (own brand)	High
	NCT02396381*† 36113,115	PMI	Parallel RCT	Ambulatory	6 months	Inhaled; ad libitum	HTP (IQOS2.2), CC (own brand)	High
	NCT01970995*† 37116,118	PMI	Parallel RCT	Confined (5 days) Ambulatory (85 days)	90 days	Inhaled; ad libitum	HTP (IQOS2.2 menthol), CC (own brand menthol), SA (smoking)	High
	NCT02466412* ^119^	PMI	Crossover RCT	Confined	Single use	Inhaled; ad libitum	HTP (CHTP1.1 menthol), CC (own brand menthol)	High
	NCT02649556*† ^38 120^	PMI	Parallel RCT	Ambulatory	12 months	Inhaled; ad libitum	HTP (IQOS2.2), CC (own brand)	High
	NCT01967732* ^48 121^	PMI	Crossover RCT	Confined	Single use	Inhaled; ad libitum	HTP (IQOS2.2), CC (own brand)	High
	NCT01780688* ^122 123^	PMI	Crossover RCT	Confined	Single use	Inhaled; ad libitum	HTP (IQOS2.1), CC (own brand)	High
	NCT01967706* 92124,126	PMI	Crossover RCT	Confined	Single use	Inhaled; ad libitum	HTP (IQOS2.2 menthol), CC (own brand), NRT (Nicorette gum)	High
	NCT03364751*† ^39 127 128^	PMI	Parallel RCT	Ambulatory	6 months	Inhaled; ad libitum	HTP (IQOS), CC (own brand)	High
**Independent**	DRKS00012919† 129,131	Medizinische Klinik III of the UKSH	Crossover RCT	Confined	Single use	Inhaled; ad libitum	HTP (IQOS), CC (Marlboro Gold), ECs (eGo-T nicotine; eGo-T no nicotine; JUUL)	High
	NCT03301129† ^132 133^	University of Roma La Sapienza	Crossover RCT	Confined	Single use	Inhaled; ad libitum	HTP (IQOS), CC (Marlboro Gold), EC (Blu Pro)	Unclear
	NCT03435562† ^134^	Virginia Commonwealth University, NIDA	Crossover RCT	Confined	Single use+90 min	Inhaled; restricted+ad libitum	HTP (IQOS), CC (own brand), EC (JUUL)	High
	NCT03452124† ^135 136^	National and Kapodistrian University of Athens	Crossover RCT	Confined	7 min	Inhaled; ad libitum	HTP (IQOS), CC (Marlboro Red)	High
	Ioakeimidis, 2021† ^137^	Athens Medical School, Hippokration Hospital	Crossover RCT	Confined	5 min	Inhaled; ad libitum	HTP (IQOS), CC (“standard tobacco cigarette”, sham CC	Unclear
	Lopez, 2016† ^138^	NIDA, Centre for Tobacco Products (FDA)	Crossover RCT	Confined	20 puffs	Inhaled; restricted	HTP (PAX), CC (own brand), EC (eGo)	High
	DRKS00020446† ^139 140^	Medizinische Klinik III of the UKSH	Crossover RCT	Confined	Single use	Inhaled; ad libitum	HTPs (IQOS; Glo), CC (Marlboro Gold), ECs (eGo no nicotine; eGo no liquid)	Unclear
	Lyytinen, 2024† ^141^	Swedish Heart-Lung foundation, Swedish Heart and Lung Association, Swedish Society of Medicine, Stockholm County Council, County Council of Västerbotten.	Crossover RCT	Confined	14 min	Inhaled; restricted	HTP (IQOS 3 Multi), ambient air	Unclear
	NCT04824495† ^142 143^	Medical University of Vienna, Swedish Heart-Lung foundation, Swedish Heart and Lung Association, Swedish Society of Medicine, Stockholm County Council, County Council of Västerbotten.	Crossover RCT	Confined	14 min	Inhaled; restricted	HTP (IQOS 3 Multi), ambient air	Unclear
	Yaman, 2021*† ^144^	Near East University, Mersin City Training and Research Hospital	Crossover RCT	Confined	5 min	Inhaled; restricted	HTP (IQOS 2.4 plus or 3 duo), CC (Marlboro Gold Light)	Unclear
Unclear	ChiCTR2200065055*^145 146^†	Beijing Great Physician Commonweal Foundation	Parallel RCT	Confined (7 days) Ambulatory (14 days)	21 days	Inhaled; ad libitum	HTP (WONZ), CC (own brand)	High

* Included in adverse event syntheses.

† Included in BoPH syntheses.

BAT, British American Tobacco; BoPH, biomarkers of potential harm; CC, combustible cigarette; CHTP, carbon heated tobacco product; EC, electronic cigarette; FDA, Food and Drug Administration; HTP, heated tobacco product; JTI, Japan Tobacco International; NIDA, National Institute on Drug Abuse; NRT, nicotine replacement therapy; Own brand, participant’s own cigarette brand of choice; PMI, Philip Morris International; RCT, randomised controlled trial; SA, smoking abstinence.

### Risk of bias

32 studies were rated high risk of bias, and unclear in the remaining eight. Risk of bias judgements for each study are provided in online supplemental table 2 and figures 2 and 3. The most common reasons for high risk of bias were inadequate blinding of participants and personnel (30 studies) and selective outcome reporting (13 studies).

Selection bias could not be assessed in^25^ because the unit of assignment was areas of skin on each participant. The Cochrane RoB tool is designed to assess trials in which individuals are the unit of assignment.

### Impact on biomarkers of potential harm

Excluding Dalrymple, 2022, 24 studies measured BoPH. 10 were independent (exposure duration range: single use—90 min; 4 high and 6 unclear risk of bias), 13 were industry-affiliated (5 days to 12 months; all high risk of bias) and 1 had unclear affiliation (21 days; high risk of bias) (table 1). From these, we extracted data on 159 unique outcomes, which measured 143 BoPH. There were more outcomes than biomarkers because some were measured in different ways, such as the same spirometry parameters being measured prebronchodilator and postbronchodilator. The biomarkers covered cardiovascular function, endothelial dysfunction, inflammation, respiratory function, metabolic syndrome (including lipid metabolism and metabolic status), oral health, oxidative stress and platelet function.

**Figure 1:**
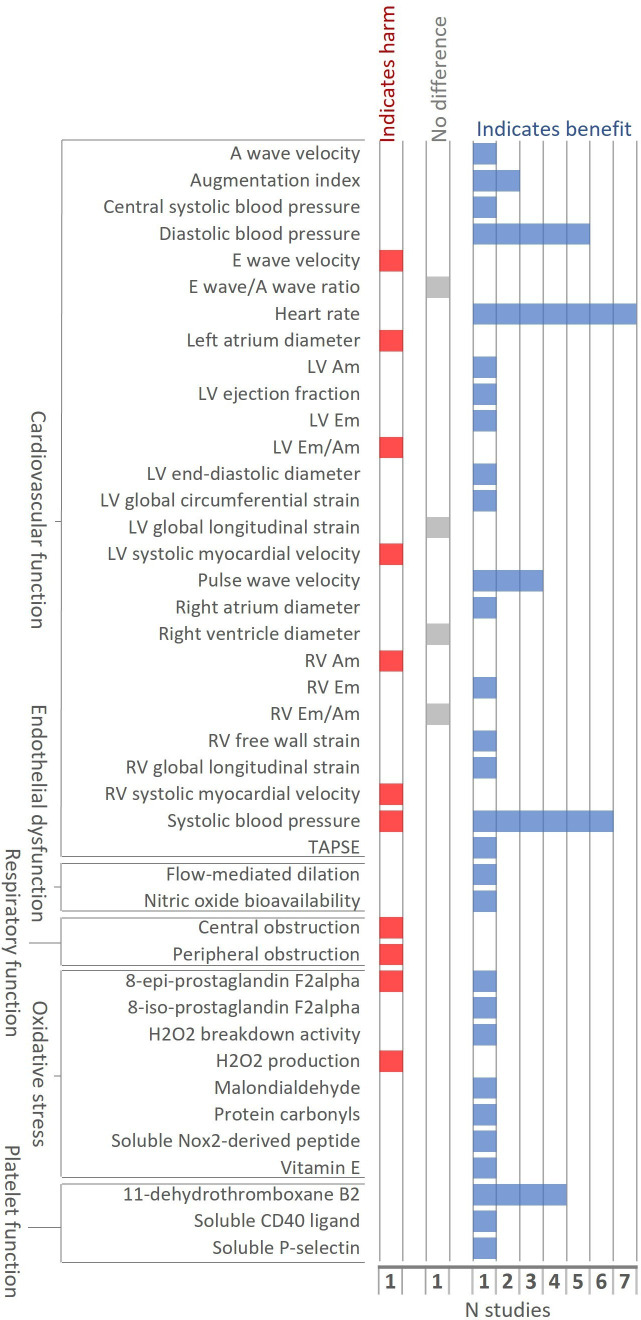
Direction of effect between HTP and cigarette arms at last follow-up in confined studies. AM, peak late diastolic velocity; EM, peak early diastolic velocity; HTP, heated tobacco product; L/R/V, left/right/ventricle; TAPSE, tricuspid annular plane systolic excursion.

11 studies measuring BoPH compared exclusive HTP and cigarette use under confined conditions. Three were industry affiliated (all 5 days; all high risk of bias) and eight were independent (single use—90 min; three high and five unclear risk of bias). Among these studies, HTPs had largely beneficial effects on biomarkers of cardiovascular function, endothelial dysfunction, oxidative stress and platelet function, compared with cigarettes (figure 1). One study found HTPs had a harmful effect on central and peripheral obstruction (respiratory function; figure 1).

**Figure 2:**
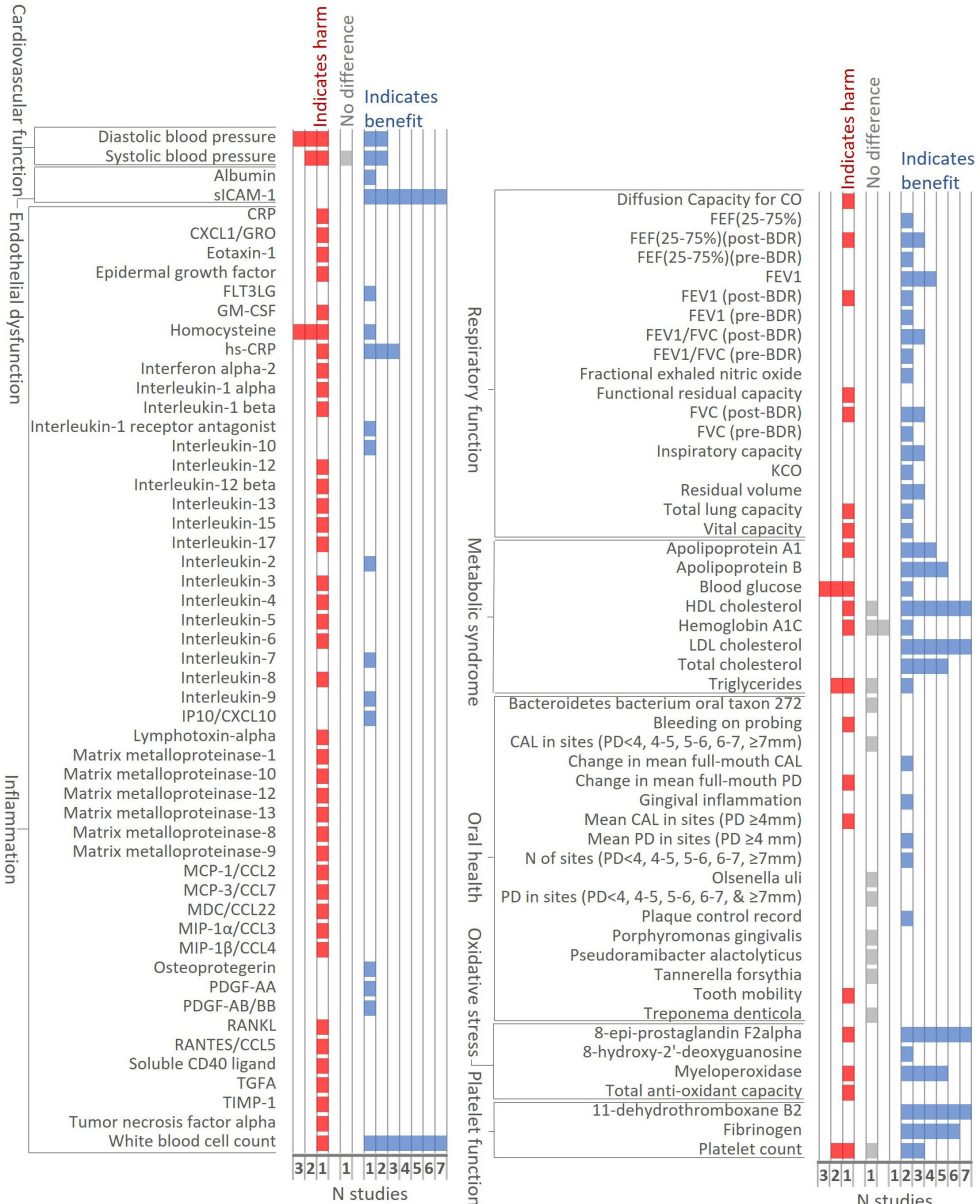
Direction of effect between HTP and cigarette arms at last follow-up in ambulatory studies. BDR, bronchodilator; CAL, clinical attachment level; CO, carbon monoxide; CXCL1/GRO, chemokine ligand 1; FEF, forced expiratory flow; FEV1, forced expiratory volume in 1 s; FLT3LG, fms-related tyrosine kinase 3 ligand; FVC, forced vital capacity; GM-CSF, granulocyte-macrophage colony-stimulating factor; HDL, high-density lipoprotein; hs-CRP, high sensitivity-C-reactive protein; HTP, heated tobacco product; IP10/CXCL10, chemokine (C-X-C motif) ligand 10; KCO, carbon monoxide transfer coefficient; LDL, low-density lipoprotein; MDC/CCL22, macrophage derived chemokine; MCP-1/CCL2, monocyte chemotactic protein-1; MCP-3/CCL7, chemokine ligand 7; MIP-1α/CCL3, macrophage inflammatory protein-1 alpha; MIP-1β/CCL4, macrophage inflammatory protein-1 beta; PD, pocket depth; PDGF-AA, platelet derived growth factor isoform AA; PDGF-AB/BB, platelet derived growth factor isoform AB/BB; RANKL, receptor activator nuclear kappa B ligand; RANTES/CCL5, regulated on activation normal T-cell expressed and secreted; TIMP-1, tissue inhibitor of metalloproteinase-1; TGFα, transforming growth factor alpha.

Nine studies compared HTPs and cigarettes under ambulatory conditions: eight industry-affiliated (90 days to 12 months; all high risk of bias) and one of unclear affiliation (21 days; high risk of bias). Data from these studies indicated HTPs had mostly beneficial effects compared with cigarettes on biomarkers of endothelial dysfunction, respiratory function, metabolic syndrome, oxidative stress and platelet function (figure 2). Directions of effect were mixed for cardiovascular function and oral health markers. While HTPs had beneficial effects on a few inflammation markers, especially white cell count, harmful effects were observed across a wide array of inflammation markers in one study (NCT03364751).

**Figure 3:**
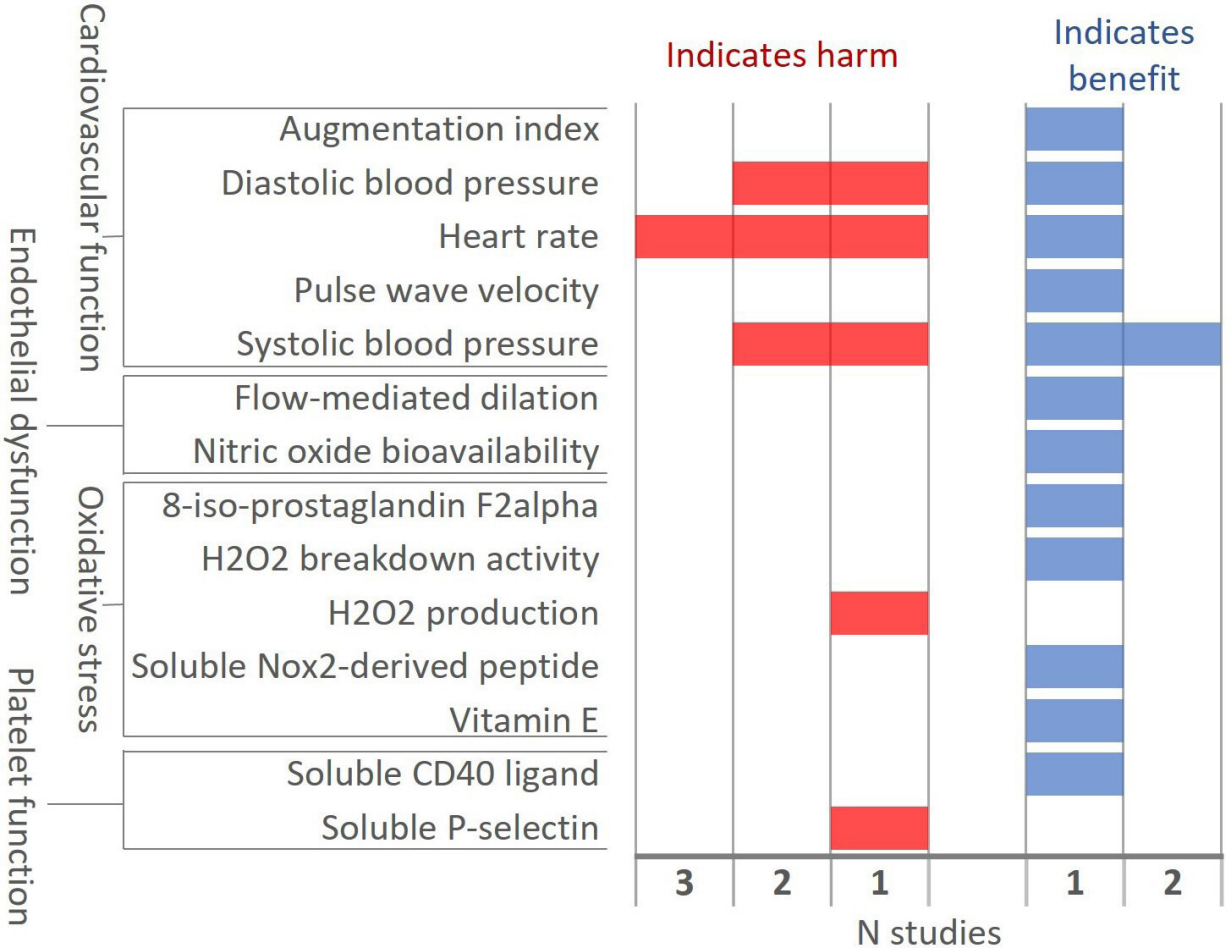
Direction of effect between HTP and e-cigarette arms at last follow-up in confined studies. HTP, heated tobacco product.

Four studies under confined conditions compared HTPs and e-cigarettes (all independent); findings were mixed (figure 3). Data from two studies (NCT03301129, single use, unclear risk of bias; and DRKS00012919, single use, high risk of bias) indicated exclusive use of HTPs had largely beneficial effects compared with e-cigarettes on biomarkers of cardiovascular function, endothelial dysfunction, oxidative stress and platelet function. However, the other two studies (NCT03435562, single use plus 90 min, high risk of bias; and Lopez 2016, single use, unclear risk of bias) indicated HTPs had harmful effects on biomarkers of cardiovascular function compared with e-cigarettes. None of the ambulatory studies included an e-cigarette arm.

**Figure 4:**
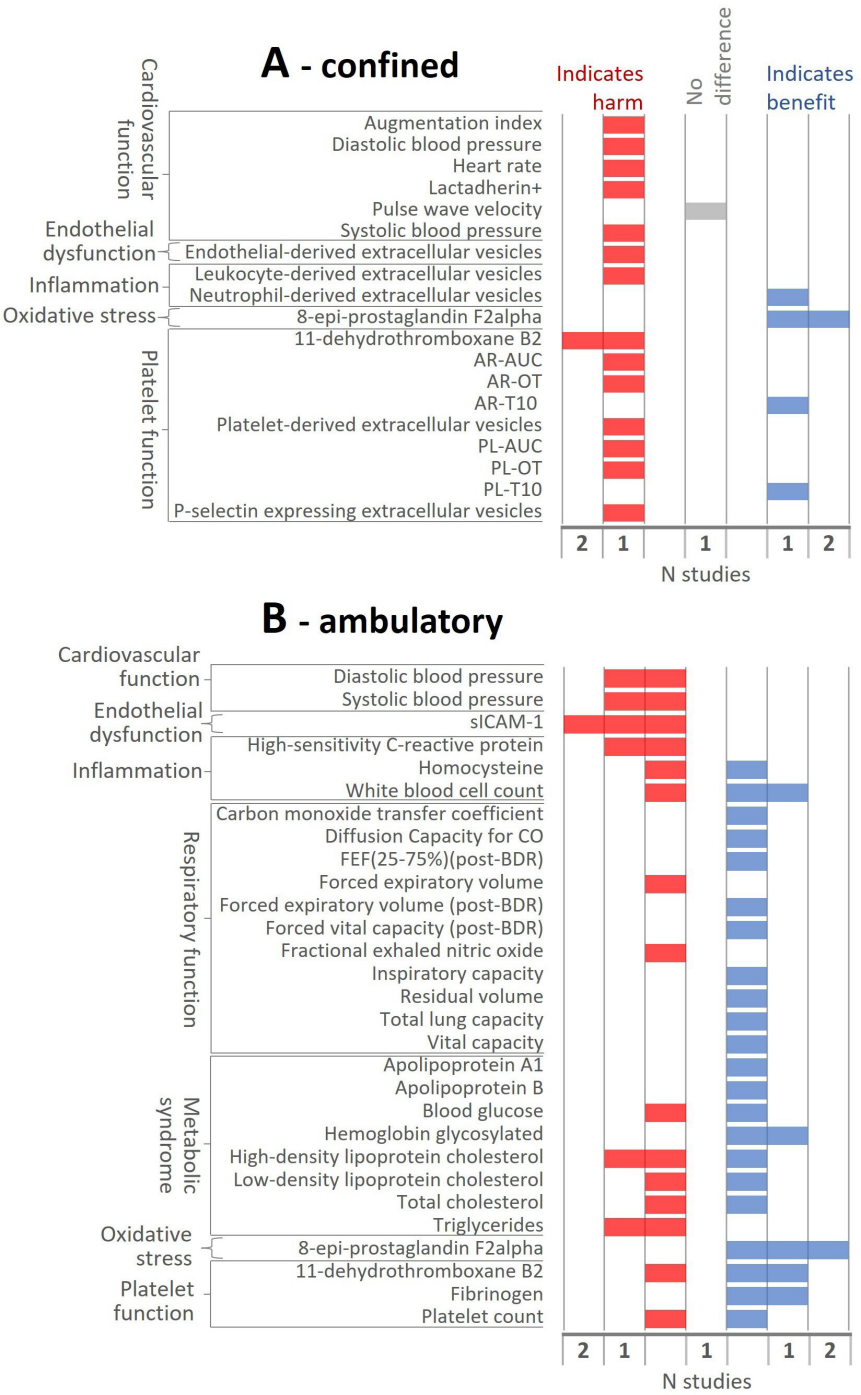
Direction of effect between HTP and smoking abstinence arms at last follow-up in (**A**) confined studies and (**B**) ambulatory studies. AR, atheroma chip; AUC, area under the curve; BDR, bronchodilator; CO, carbon monoxide; FEF, forced expiratory flow; HTP, heated tobacco product; OT, time to reach occlusion pressure in microchip; PL, platelet chip; T10, time to reach start of occlusion, meaning 10 kPa pressure in microchip.

Four studies compared HTPs with smoking abstinence under confined conditions: two industry-affiliated (both 5 days; high risk of bias) and two independent (both 14 min; unclear risk of bias). Data from these indicate exclusive HTP use had a beneficial effect on an oxidative stress biomarker, but largely harmful effects on cardiovascular function, endothelial dysfunction and platelet function (figure 4A). Contrasting effects were observed for two inflammation biomarkers. Three studies also compared HTPs to smoking abstinence under ambulatory conditions (all industry-affiliated and high risk of bias; ISRCTN81075760, NCT01970995, NCT01989156). Directions of effect were mixed both within and across studies, especially for biomarkers of inflammation and metabolic syndrome (figure 4B).

We also synthesised the effect HTPs had on BoPH over time in smokers assigned to use HTPs (ie, within arm analyses of baseline vs last follow-up in HTP arms). Exclusive HTP use in smokers under confined settings had mostly harmful effects on biomarkers, namely markers of cardiovascular function, endothelial dysfunction, inflammation, respiratory function, oxidative stress and platelet function (online supplemental figure 4). Under ambulatory conditions, HTPs had largely beneficial effects on markers of endothelial dysfunction, oral health, oxidative stress and metabolic syndrome in smokers (online supplemental figure 5). However, they had mixed effects on cardiovascular function, inflammation, and platelet function, and largely harmful effects on respiratory function.

### Impact on adverse events

#### Adverse events

28 studies reported adverse event data, all of which were industry-affiliated bar one (ChiCTR2200065055) (table 1). 15 studies were ineligible for meta-analysis. This was due to crossover design for 11 studies. Data from these are reported in online supplemental table 3. A further four were ineligible due to reporting zero adverse events in any group (UMIN000045304, UMIN000025777, UMIN000041539). ISRCTN81075760 was ineligible for the meta-analysis as data were not reported separately for each arm.

13 studies were included in the meta-analysis (5 days to 12 months; all high risk of bias). The overall rate of participants reporting adverse events was not statistically significantly different between HTP and: cigarette groups (RR 1.01, 95% CI 0.87 to 1.16; I^2^=45%; 2804 participants across 13 studies; online supplemental figure 6A); smoking abstinence groups (RR 1.04, 95% CI 0.86 to 1.36; I^2^=0%; 681 participants across 6 studies; online supplemental figure 6B); and e-cigarette groups (RR 0.81, 95% CI 0.38 to 1.73; I^2^=N/A; 84 participants in 1 study; online supplemental figure 6C). This was true regardless of whether the studies were conducted in confined or ambulatory settings.

Across all studies (included and excluded from the meta-analysis), adverse events in HTP groups commonly included headache, cough and presyncope. Most were mild or moderate in severity. The following events were noted by study authors as possibly related to HTP use: dry mouth, headache, increased weight, salivary hypersecretion, abnormal spirometry, sneezing, upper airway cough syndrome, non-cardiac chest pain, cough, wheezing, syncope, vertigo, oral pain, oropharyngeal pain, hypertriglyceridaemia, musculoskeletal chest pain, myocardial ischaemia, hyperhidrosis, hypertension, nausea and proteinuria.

#### Serious adverse events

Serious adverse events were reported in 10 studies, which were all industry-affiliated. Three were ineligible due to cross-over design. Data from these are reported in online supplemental table 3. UMIN000045304 and ChiCTR2200065055 were ineligible for reporting zero adverse events in any group and ISRCTN81075760 due to insufficient data. In the latter, nine serious adverse events were reported by day 360 across 370 participants. The authors provided no other details but considered them unrelated to any study product.

Five studies were included in the meta-analysis (5 days to 12 months; all high risk of bias). The overall rate of participants reporting serious adverse events was not statistically significantly different between HTP and cigarette groups (RR 1.15, 95% CI 0.65 to 2.05; I^2^=0%; 2005 participants across 5 studies; online supplemental figure 7A). ISRCTN80651909 (5 days; high risk of bias) was the only study to report serious adverse event data and include e-cigarette or smoking abstinence arms. The overall rate of participants reporting serious adverse events was not statistically significantly different between HTP and smoking abstinence (RR 1.50, 95% CI 0.06 to 35.73; I^2^=N/A; 88 participants in 1 study; online supplemental figure 7B) or e-cigarette groups (RR 1.55, 95% CI 0.07 to 36.94; I^2^=N/A; 89 participants in 1 study; online supplemental figure 7C).

In participants assigned HTPs, the following serious adverse events were reported:

- NCT02649556 reported: appendicitis with peritonitis, epiglottitis, influenza, mycoplasmal pneumonia, head injury with seizure, hip fracture, laceration, foot fracture, costochondritis, completed suicide, menorrhagia, pneumonia aspiration. It was not reported whether these were product related.
- NCT02396381 reported: mycoplasmal pneumonia, head injury with seizure, laceration, metastases to small intestine with anaemia, completed suicide and alcohol abuse. It was not reported whether these were considered product related.
- NCT02641587 reported appendicitis and peritonsillar abscess. These were not considered product related.
- ISRCTN80651909 reported decompensated tachycardia. This was not considered product related.

### Novel skin staining study (Dalrymple, 2022)

This study was industry-affiliated, judged to be at unclear risk of bias and conducted under confined conditions. Areas of non-smokers’ skin were exposed to product emissions generated by a smoking machine. Five outcomes related to oxidative stress were measured: catalase, malondialdehyde, squalene, squalene monohydroperoxide and squalene monohydroperoxide/squalene ratio. Compared with the control (an unexposed area of skin), HTP emissions had harmful effects on all these outcomes. HTP emissions also had harmful effects compared with e-cigarette emissions on all but one outcome (squalene monohydroperoxide). Compared with cigarette smoke, HTP emissions had beneficial effects on all the outcomes.

## Discussion

This is the first independent and most comprehensive systematic review of the available data on the effects of HTPs on BoPH and adverse events. In line with our previous review,^11^ most of the 40 interventional clinical trials included were industry-affiliated and at high risk of bias. The studies were notably short; the longest being only 12 months, but most 5 days or less. Few studies included e-cigarette or smoking abstinence arms, instead comparing HTPs with cigarettes. Despite emerging evidence of HTP use among non-smokers,^26^,^28^ only one, notably different, study assessed the impact of HTPs in non-smokers. This likely reflects the ethical complexities of assigning an addictive product to non-smokers, in which it can only do harm.

We collated data on 143 BoPH from 24 studies. Overall, the findings are so mixed that these data provide no clear indication of the relative risks or benefits of HTPs, including insufficient evidence to indicate any certain benefits over cigarettes. Beneficial and harmful effects were observed under both confined and ambulatory settings. The limited beneficial effects observed in smokers following exclusive HTP use for a short period (up to 5 days) were not consistently replicated under longer, ambulatory settings. Mixed effects were also apparent when comparing HTPs to e-cigarettes or smoking abstinence. Meta-analysis revealed HTP users reported no fewer adverse events than participants assigned comparators. Our findings align with those from observational clinical studies to date, which have found HTP users have improved levels of some BoPH compared with smokers, but this is not ubiquitous across all BoPH and, compared with non-smokers, some BoPH worsen.^29^,^31^

The variance in direction of effects may be attributable to differences in study design, the specific makes and models of products used, or the presence of conflicts of interest. For example, analysis of broader HTP literature revealed industry-affiliated papers were significantly more likely to conclude HTPs were more desirable than cigarettes.^32^

We identified some unexpected directions of effect, most likely due to chance given the short trial durations and direction of effect syntheses not accounting for significance or magnitude of effects. Unusual and differing effects may also be explained by continued cigarette use among those assigned to other interventions, which would be particularly apparent when comparing confined and ambulatory settings. Indeed, all of the studies that included an ambulatory period reported some level of this phenomenon.^33^,^39^ This is consistent with growing evidence documenting high prevalence of dual or poly use of HTPs and other products, especially cigarettes, among real-world users.^40^,^42^ Effects observed in smokers could also be impacted by the residual effects of long-term smoking^43 44^ or changes in product use behaviour under trial conditions. The latter was observed in several of the studies.^33^,^3745^ Moreover, biomarkers which increase in one study yet decrease in another may be explained through pretrial abstinence or wash-out periods; this may be particularly apparent in cross-over trials. In regard to no differences in adverse events, the picture may be complicated by the fact that cigarette withdrawal is associated with a range of temporary, non-serious adverse events.^49^

There is no set of validated biomarkers with which to predict the risk of developing tobacco-related diseases.^50^ Most BoPH used in these studies indicate broad systemic effects; few disease-specific (notably cancer-related) biomarkers were used.^51^ Further, the studies are simply too short for the manifestation of tobacco-related diseases, such as chronic obstructive pulmonary disease, which develops over decades.^52^

We identified instances of selective reporting, particularly among industry-affiliated trials, raising concerns that vital data may not be being reported. This is particularly salient in light of the US marketing order renewal for IQOS, which permits PMI to market IQOS as a ‘modified risk tobacco product’. In its previous submissions to the FDA, PMI’s study reports were made public, making otherwise unpublished clinical data available for independent reviews, like ours. But for the renewal, reports were redacted.^10^ Once these and data from ongoing trials (especially any at low risk of bias) are made available, it would be pertinent to update our review. For now, the lack of validated biomarkers and long-term evidence, in conjunction with a poor-quality and potentially incomplete data set, hinders understanding of potential health risks of HTPs.

### Strengths and limitations

This is the first review to systematically synthesise all available BoPH and adverse event data from interventional clinical trials investigating HTPs. Despite both being measured in many of the included studies, we prioritised BoPH over biomarkers of exposure because the latter have been reviewed previously^8 9 13 14^ and are generally not directly indicative of physiological changes or disease development.^19^ We reviewed the largest quantity of BoPH data on HTPs to date, partly attributable to including non-peer-reviewed materials. However, limitations exist. First, due to the availability and heterogeneity of the data, we could not use meta-analyses to analyse BoPH data. Second, most studies were at high risk of bias, and our previous review highlighted the prevalence of substandard conduct and reporting.^11^ We also cannot rule out publication bias, especially in studies for which no a priori protocol is available. Therefore, inferences of the health effects based on these studies should not be considered conclusive findings. Even if the studies were at low risk of bias, this would remain difficult using BoPH and adverse event data alone, given these are surrogate markers, not definitive clinical endpoints.

## Conclusions

A growing quantity of adverse event and BoPH data are available from clinical trials investigating HTPs. However, determining the health impacts of HTPs from these data is significantly hindered by high risk of bias, heterogeneity, unrealistic study conditions, very short durations and tobacco industry dominance. Bearing these caveats in mind, the existing data indicate HTPs have the potential to be harmful to both smokers and non-smokers, and that potential benefits in smokers switching to HTPs may be restricted to a limited subset of biomarkers whose clinical relevance is unclear. It is vital policy-makers and consumers making potentially life-changing decisions on the existing evidence are aware of its current limitations and lack of conclusiveness about the risks of HTPs. The presence of other products—both pharmaceutical and non-tobacco consumer products—for which evidence already demonstrates reduced risk profiles and greater effectiveness for smoking cessation raises critical questions about whether there is even a place for HTPs in the consumer market.

Longer, unbiased clinical trials and epidemiological studies are needed to investigate the: long-term impacts of HTP use in real-world conditions; health impacts of HTPs in the growing numbers of non-smokers using them; and relative effects of HTPs and other interventions, especially competitive products already available to consumers and proven to aid smoking cessation. Given emerging evidence that the tobacco industry continues to engage in problematic scientific practices,^53^,^56^ such studies are unlikely to emerge unless regulators require them or alternative systems for funding such research are developed.^53^

## Supplementary material

10.1136/tc-2024-059000 online supplemental file 1

## Data Availability

All data relevant to the study are included in the article or uploaded as supplementary information.
