# Supplementary material for: Impact of heated tobacco products on biomarkers of potential harm and adverse events: a systematic review and meta-analysis
Source: Tob Control. 2025 Apr 29;35(3):e059000. doi: 10.1136/tc-2024-059000 (PMC13217111; doi:10.1136/tc-2024-059000)

**The impact of heated tobacco products on biomarkers of potential harm and adverse events: a systematic review and meta-analysis**

*Supplementary Materials*

Contents

[Supplementary Appendix 1. Coding of trial affiliation. 2](#_Toc190072974)

[Supplementary Table 1. Direction of effect indicative of harm for each biomarker, alongside supporting literature in addition to the reporting in the original study. 3](#_Toc190072975)

[Supplementary Figure 1. PRISMA flow diagram for study selection. Abbreviations: HTP = heated tobacco; BoPH = biomarkers of potential harm; AE = adverse events. 9](#_Toc190072976)

[Supplementary Table 2. Risk of bias judgements and support for judgements for each included study. 10](#_Toc190072977)

[Supplementary Figure 2. Summary plot showing risk of bias judgements across studies. 23](#_Toc190072978)

[Supplementary Figure 3. Risk of bias traffic light plot: review authors’ judgments about risk of bias items for each included study. 24](#_Toc190072979)

[Supplementary Figure 4. Direction of effect between baseline and last follow-up in HTP arms in confined studies. 25](#_Toc190072980)

[Supplementary Figure 5. Direction of effect between baseline and last follow-up in HTP arms in ambulatory studies. 26](#_Toc190072981)

[Supplementary Table 3. Serious and non-serious adverse event data from crossover trials. 27](#_Toc190072982)

[Supplementary Figure 6. Effect of heated tobacco product use compared with cigarette use (A), smoking abstinence (B) and e-cigarette use (C) on rate of participants reporting adverse events. 31](#_Toc190072983)

[Supplementary Figure 7. Effect of heated tobacco product use compared with cigarette use (A), smoking abstinence (B) and e-cigarette use (C) on rate of participants reporting serious adverse events. 32](#_Toc190072984)

# Supplementary Appendix 1. Coding of trial affiliation.

Trials were coded as ‘Industry-affiliated’ if:

- the study sponsor named on the trial registration was a tobacco company or other organisation directly funded by a tobacco company; or
- funding statements in any of the trial literature indicated the trial was funded in part or in whole by a tobacco company or other organisation directly funded by a tobacco company; or
- author affiliations or conflict of interest statements indicated any author was an employee or funded by a tobacco company or other organisation directly funded by a tobacco company at the time of the trial.

Trials were coded as ‘Independent’ if:

- the sponsor named on the trial registration had no known ties to the tobacco industry; and
- funding statements in any of the trial literature indicated the trial was not funded by a tobacco company or other organisation funded by a tobacco company; and
- author affiliations and conflict of interest statements indicated authors had no contemporary (i.e., while the study was being conducted, up to and including publication) ties to the tobacco industry.

Trials were coded as ‘Unclear’ if:

- There was insufficient information to determine affiliation; or
- Reviewers could not reach consensus.

In addition to conflict of interest and funding statements provided in the trial literature, we further investigated known ties and funding using the Tobacco Tactics website (www.tobaccotactics.org), relevant literature published by the Tobacco Control Research Group (University of Bath), and conflict of interest and funding statements in other contemporary work of the authors of included studies.

# Supplementary Table 1. Direction of effect indicative of harm for each biomarker, alongside supporting literature in addition to the reporting in the original study.

|  | **Biomarker** | **Direction indicating harm** | **Additional supporting literature** |
| --- | --- | --- | --- |
| Cardiovascular function | A wave velocity | ­↑ | [https://doi.org/10.1111/echo.14514; https://doi.org/10.1016/j.jsha.2013.03.003](https://doi.org/10.1111/echo.14514) |
|  | Arterial Blood Pressure | ­↑ | https://doi.org/10.1093/ntr/ntx273 |
|  | Augmentation index | ­↑ | [https://doi.org/10.1093/eurheartj/ehq024; https://doi.org/10.1161/CIRCULATIONAHA.104.482570](https://doi.org/10.1093/eurheartj/ehq024) |
|  | Central pulse pressure | ­↑ | https://doi.org/10.1093/ntr/ntx273; https://www.ncbi.nlm.nih.gov/books/NBK482408/; https://doi.org/10.1093/eurheartj/ehq024 |
|  | Diastolic blood pressure | ­↑ | https://doi.org/10.1093/ntr/ntx273 |
|  | E wave velocity | ↓ | [https://doi.org/10.1111/echo.14514; https://doi.org/10.1016/j.jsha.2013.03.003](https://doi.org/10.1111/echo.14514) |
|  | E wave/A wave ratio | ↓ | [https://brieflands.com/articles/ircrj-132828.pdf; https://doi.org/10.1111/echo.14514; https://doi.org/10.1016/j.jsha.2013.03.003](https://brieflands.com/articles/ircrj-132828.pdf) |
|  | Heart rate | ­↑ | https://doi.org/10.1093/ntr/ntx273 |
|  | Left atrium diameter | ­↑ | <https://doi.org/10.1111/echo.14514> |
|  | Left ventricle early/late diastolic mitral annulus tissue Doppler velocities ratio | ↓ | <https://doi.org/10.1111/echo.14514> |
|  | Left ventricle ejection fraction | ↓ | <https://brieflands.com/articles/ircrj-132828.pdf> |
|  | Left ventricle global circumferential strain | ↓ | <https://brieflands.com/articles/ircrj-132828.pdf> |
|  | Left ventricle global longitudinal strain | ↓ | <https://doi.org/10.1111/echo.14514> |
|  | Left ventricle peak early diastolic velocity | ↓ | [https://brieflands.com/articles/ircrj-132828.pdf; https://doi.org/10.1111/echo.14514](https://brieflands.com/articles/ircrj-132828.pdf) |
|  | Left ventricle peak late diastolic velocity | ­↑ | <https://doi.org/10.1111/echo.14514> |
|  | Left ventricle systolic myocardial velocity | ­↑ | <https://doi.org/10.1111/echo.14514> |
|  | Left ventricular end-diastolic diameter | ­↑ | <https://doi.org/10.1111/echo.14514> |
|  | Pulse wave velocity | ­↑ | https://doi.org/10.1016/j.jacc.2007.10.065; https://doi.org/10.1161/CIRCULATIONAHA.105.555235; https://doi.org/10.1161/CIRCULATIONAHA.104.482570 |
|  | Right atrium diameter | ­↑ | <https://doi.org/10.1111/echo.14514> |
|  | Right ventricle diameter | ­↑ | <https://doi.org/10.1111/echo.14514> |
|  | Right ventricle early/late diastolic mitral annulus tissue Doppler velocities ratio | ↓ | <https://doi.org/10.1111/echo.14514> |
|  | Right ventricle free wall strain | ↓ | <https://doi.org/10.1111/echo.14514> |
|  | Right ventricle global longitudinal strain | ↓ | <https://doi.org/10.1111/echo.14514> |
|  | Right ventricle peak early diastolic velocity | ↓ | <https://doi.org/10.1111/echo.14514> |
|  | Right ventricle peak late diastolic velocity | ­↑ | <https://doi.org/10.1111/echo.14514> |
|  | Right ventricle systolic myocardial velocity | ­↑ | <https://doi.org/10.1111/echo.14514> |
|  | Systolic blood pressure | ­↑ | https://doi.org/10.1093/ntr/ntx273; https://doi.org/10.1093/eurheartj/ehq024 |
|  | Total extracellular vesicles | ­↑ | https://doi.org/10.3389/fcvm.2022.907457; https://doi.org/10.1161/CIRCRESAHA.117.310752 |
|  | Total Peripheral Resistance | ­↑ | [https://doi.org/10.1161/01.HYP.28.1.37; https://www.ncbi.nlm.nih.gov/books/NBK538308/](https://doi.org/10.1161/01.HYP.28.1.37) |
|  | Tricuspid annular plane systolic excursion | ↓ | [https://brieflands.com/articles/ircrj-132828.pdf; https://doi.org/10.1016/j.jsha.2013.03.003](https://brieflands.com/articles/ircrj-132828.pdf) |
| Endothelial dysfunction | Albumin (in urine) | ­↑ | https://doi.org/10.1161/CIRCULATIONAHA.104.482570; https://doi.org/10.1080/1354750X.2017.1419284 |
|  | Endothelial-derived extracellular vesicles | ­↑ | https://doi.org/10.1093/ntr/ntx273 |
|  | Flow-mediated dilation | ↓ | https://doi.org/10.1093/ntr/ntx273 |
|  | Nitric oxide bioavailability | ↓ | https://doi.org/10.1161/01.CIR.0000153339.27064.14; https://doi.org/10.1016/j.ccc.2019.12.009; https://doi.org/10.3390/molecules27227921 |
|  | Soluble intercellular adhesion molecule-1 | ­↑ | https://doi.org/10.1093/ntr/ntx273; https://doi.org/10.1080/1354750X.2017.1419284 |
| Inflammation | Chemokine (C-C motif) ligand 7 | ­↑ | [https://doi.org/10.1172/jci.insight.132048; https://doi.org/10.1152/ajplung.00074.2015; https://doi.org/10.1016/j.cyto.2023.156403](https://doi.org/10.1172/jci.insight.132048;) |
|  | Chemokine (C-X-C motif) ligand 1 | ­↑ | [https://doi.org/10.1002/jlb.59.1.67; https://doi.org/10.1152/ajplung.00074.2015](https://doi.org/10.1002/jlb.59.1.67;) |
|  | Chemokine (C-X-C motif) ligand 10 | ↓ | [https://doi.org/10.1152/ajplung.00074.2015; https://doi.org/10.1158/1078-0432.ccr-19-1769; https://doi.org/10.1016/j.rmed.2019.105822; https://urn.fi/URN:NBN:fi-fe2023042438477](https://doi.org/10.1152/ajplung.00074.2015;) |
|  | C-reactive protein | ­↑ | https://doi.org/10.1093/ntr/ntx273; https://doi.org/10.1080/1354750X.2017.1419284 |
|  | Eotaxin-1 | ­↑ | [https://doi.org/10.1152/ajplung.00074.2015; https://doi.org/10.1080/02770900902846349; https://doi.org/10.4049/jimmunol.178.8.5321](https://doi.org/10.1152/ajplung.00074.2015;) |
|  | Epidermal growth factor (saliva) | ↓ | [https://doi.org/10.1152/ajpgi.2000.278.1.g10; https://doi.org/10.1016/0300-483x(92)90153-6](https://doi.org/10.1152/ajpgi.2000.278.1.g10;) |
|  | Fms-related tyrosine kinase 3 ligand | ­↑ | https://doi.org/10.3389/fimmu.2024.1416870; https://doi.org/10.1183/13993003.00277-2024 |
|  | Granulocyte-macrophage colony-stimulating factor | ­↑ | [https://doi.org/10.1016/j.jaut.2009.12.003; https://doi.org/10.1152/ajplung.00074.2015](https://doi.org/10.1016/j.jaut.2009.12.003) |
|  | High-sensitivity C-reactive protein | ­↑ | [https://doi.org/10.1093/ntr/ntx273; https://doi.org/10.1080/1354750X.2017.1419284](https://doi.org/10.1093/ntr/ntx273) |
|  | Homocysteine | ­↑ | [https://doi.org/10.1001/jama.288.16.2015; https://doi.org/10.1080/1354750X.2017.1419284](https://doi.org/10.1001/jama.288.16.2015) |
|  | Interferon alpha-2 | ­↑ | <https://doi.org/10.3390/biomedicines12040748> |
|  | Interferon-gamma | ↓ | [https://doi.org/10.1067/mai.2000.107751; https://doi.org/10.21037/tlcr.2019.03.02](https://doi.org/10.1067/mai.2000.107751) |
|  | Interleukin-1 alpha | ­↑ | https://doi.org/10.1152/ajplung.00074.2015; https://doi.org/10.5021/ad.2014.26.1.11 |
|  | Interleukin-1 beta | ­↑ | [https://doi.org/10.3390/molecules27123715; https://doi.org/10.1067/mai.2000.107751; https://doi.org/10.1152/ajplung.00074.2015](https://doi.org/10.3390/molecules27123715;) |
|  | Interleukin-1 receptor antagonist | ­↑ | https://www.europeanreview.org/article/7005; https://doi.org/10.2337/dc08-1161 |
|  | Interleukin-10 | ↓ | https://doi.org/10.7150/ijms.13800; https://doi.org/10.1155/2014/158530; https://doi.org/10.1152/ajplung.00074.2015; https://doi.org/10.1016/j.jaut.2009.12.003 |
|  | Interleukin-12 | ­↑ | https://doi.org/10.3109/08958378.2015.1013227; https://doi.org/10.1155/2014/158530 |
|  | Interleukin-12 beta | ↓ | https://doi.org/10.4049/jimmunol.181.2.1536; https://doi.org/10.1038/s41598-021-91510-x |
|  | Interleukin-13 | ­↑ | [https://doi.org/10.3390/diseases12070144; http://dx.doi.org/10.1165/rcmb.2009-0117OC](https://doi.org/10.3390/diseases12070144) |
|  | Interleukin-15 | ­↑ | <https://doi.org/10.1186/s12903-022-02700-6> |
|  | Interleukin-17 | ­↑ | <https://doi.org/10.3390/diseases12070144> |
|  | Interleukin-2 | ↓ | [https://doi.org/10.1067/mai.2000.107751; https://doi.org/10.1152/ajplung.00074.2015](https://doi.org/10.1067/mai.2000.107751) |
|  | Interleukin-3 | ­↑ | https://doi.org/10.1084/jem.20180722; https://doi.org/10.3389/fimmu.2024.1411047 |
|  | Interleukin-4 | ­↑ | [https://doi.org/10.1152/ajplung.00074.2015; https://doi.org/10.1111/j.1365-2249.1994.tb06533.x](https://doi.org/10.1152/ajplung.00074.2015) |
|  | Interleukin-5 | ­↑ | <https://pubmed.ncbi.nlm.nih.gov/24706315/> |
|  | Interleukin-6 | ­↑ | [https://doi.org/10.1016/j.jaut.2009.12.003; https://doi.org/10.1093/ntr/ntx273; https://doi.org/10.1152/ajplung.00074.2015](https://doi.org/10.1016/j.jaut.2009.12.003;) |
|  | Interleukin-7 | ↓ | [https://doi.org/10.1152/ajplung.00074.2015; http://dx.doi.org/10.1186/1471-2431-13-57; https://doi.org/10.1016/j.sjbs.2019.11.001](https://doi.org/10.1152/ajplung.00074.2015) |
|  | Interleukin-8 | ­↑ | [https://doi.org/10.1016/j.jaut.2009.12.003; https://doi.org/10.1038/s41598-020-68753-1](https://doi.org/10.1016/j.jaut.2009.12.003) |
|  | Interleukin-9 | ↓ | [https://doi.org/10.3390/biomedicines12040748; https://doi.org/10.1183/23120541.00639-2021](https://doi.org/10.3390/biomedicines12040748;) |
|  | Leukocyte-derived extracellular vesicles | ­↑ | [https://doi.org/10.1155/2018/4692081; https://doi.org/10.3390/ijms25010388](https://doi.org/10.1155/2018/4692081) |
|  | Lymphotoxin-alpha | ­↑ | <https://doi.org/10.1152/ajplung.00074.2015> |
|  | Macrophage inflammatory protein-1 alpha | ­↑ | [https://doi.org/10.1152/ajplung.00074.2015; https://doi.org/10.3109/01902149409031733; https://doi.org/10.1007/s00262-006-0149-3](https://doi.org/10.1152/ajplung.00074.2015) |
|  | Macrophage inflammatory protein-1 beta | ­↑ | [https://doi.org/10.1007/s00262-006-0149-3; https://doi.org/10.1152/ajplung.00074.2015](https://doi.org/10.1007/s00262-006-0149-3;) |
|  | Macrophage-derived chemokine | ­↑ | https://doi.org/10.1152/ajplung.00074.2015; https://doi.org/10.1016/j.clim.2005.03.001; https://doi.org/10.1038/s41598-020-68753-1 |
|  | Matrix metalloproteinase-1 | ­↑ | [https://doi.org/10.1161/01.ATV.0000199268.27395.4f; https://doi.org/10.7150/ijms.79889](https://doi.org/10.1161/01.ATV.0000199268.27395.4f) |
|  | Matrix metalloproteinase-10 | ­↑ | https://doi.org/10.1161/01.ATV.0000199268.27395.4f |
|  | Matrix metalloproteinase-12 | ­↑ | https://doi.org/10.1161/01.ATV.0000199268.27395.4f |
|  | Matrix metalloproteinase-13 | ­↑ | https://doi.org/10.1161/01.ATV.0000199268.27395.4f |
|  | Matrix metalloproteinase-8 | ­↑ | https://doi.org/10.1161/01.ATV.0000199268.27395.4f |
|  | Matrix metalloproteinase-9 | ­↑ | [https://doi.org/10.1152/ajplung.00074.2015; https://doi.org/10.4274/MMJ.galenos.2022.45057](https://doi.org/10.1152/ajplung.00074.2015) |
|  | Monocyte chemotactic protein-1 | ­↑ | [https://doi.org/10.3109/08958378.2015.1013227; https://doi.org/10.4274/MMJ.galenos.2022.45057](https://doi.org/10.3109/08958378.2015.1013227) |
|  | Neutrophil-derived extracellular vesicles | ­↑ | [https://doi.org/10.1155/2018/4692081; https://doi.org/10.3390/ijms25010388](https://doi.org/10.1155/2018/4692081) |
|  | Osteoprotegerin | ↓ | [https://doi.org/10.1016/j.archoralbio.2020.104714; https://doi.org/10.1111/j.1600-051x.2007.01048.x; https://doi.org/10.4103/2277-9175.180992; https://doi.org/10.3390/jcm8030406](https://doi.org/10.1016/j.archoralbio.2020.104714) |
|  | Platelet derived growth factor isoform AA | ↓ | [https://doi.org/10.1164/rccm.200605-585OC; https://doi.org/10.1007/s10006-025-01345-3; https://doi.org/10.3892/etm.2019.8025](https://doi.org/10.1164/rccm.200605-585OC) |
|  | Platelet derived growth factor isoform AB/BB | ↓ | https://doi.org/10.1164/rccm.200605-585OC; https://doi.org/10.1007/s10006-025-01345-3; https://doi.org/10.3892/etm.2019.8025 |
|  | Receptor activator nuclear kappa B ligand | ­↑ | <https://doi.org/10.1155/2014/731039> |
|  | Regulated upon activation normal T-cell expressed and secreted | ­↑ | https://doi.org/10.1152/ajplung.00074.2015; https://doi.org/10.1093/toxsci/kfj147; https://doi.org/10.4049/jimmunol.166.1.552 |
|  | Tissue inhibitor of metalloproteinase-1 | ­↑ | <https://doi.org/10.4274/MMJ.galenos.2022.45057> |
|  | Transforming growth factor alpha | ­↑ | <https://doi.org/10.1002/jat.4469> |
|  | Tumor necrosis factor alpha | ­↑ | [https://doi.org/10.1016/j.jaut.2009.12.003; https://doi.org/10.1093/ntr/ntx273; https://doi.org/10.3390/diseases12070144](https://doi.org/10.1016/j.jaut.2009.12.003) |
|  | White blood cell count | ­↑ | [https://doi.org/10.3109/08958378.2015.1013227; https://doi.org/10.1080/1354750X.2017.1419284](https://doi.org/10.3109/08958378.2015.1013227) |
| Respiratory function | Carbon monoxide transfer coefficient | ↓ | [https://www.ncbi.nlm.nih.gov/pmc/articles/PMC3229853/; https://www.ncbi.nlm.nih.gov/books/NBK556149/](https://www.ncbi.nlm.nih.gov/pmc/articles/PMC3229853/) |
|  | Central obstruction | ­↑ | [https://doi.org/10.1016/j.rmed.2003.09.005; https://doi.org/10.4103/0970-2113.184875; https://doi.org/10.1016/S0140-6736(22)00470-6](https://doi.org/10.1016/j.rmed.2003.09.005) |
|  | Diffusion Capacity for CO | ↓ | [https://pmc.ncbi.nlm.nih.gov/articles/PMC3229853/; https://www.ncbi.nlm.nih.gov/books/NBK556149/](https://pmc.ncbi.nlm.nih.gov/articles/PMC3229853/) |
|  | Forced expiratory flow at 25–75% of forced vital capacity | ↓ | https://pubmed.ncbi.nlm.nih.gov/12841492/ |
|  | Forced expiratory volume | ↓ | https://doi.org/10.1093/ntr/ntx273; https://doi.org/10.1080/1354750X.2017.1419284 |
|  | Forced expiratory volume in the first one second/forced vital capacity | ↓ | [https://pmc.ncbi.nlm.nih.gov/articles/PMC3229853/; https://pubmed.ncbi.nlm.nih.gov/12841492/](https://pmc.ncbi.nlm.nih.gov/articles/PMC3229853/) |
|  | Forced vital capacity | ↓ | [https://pmc.ncbi.nlm.nih.gov/articles/PMC3229853/; https://pubmed.ncbi.nlm.nih.gov/12841492/](https://pmc.ncbi.nlm.nih.gov/articles/PMC3229853/) |
|  | Fractional exhaled nitric oxide | ↓ | [https://doi.org/10.1136/thorax.58.2.175; https://doi.org/10.1080/00039890009604040; https://doi.org/10.5402/2011/832560; https://doi.org/10.1183/09031936.06.00113705](https://doi.org/10.1136/thorax.58.2.175;) |
|  | Functional residual capacity | ↓ | <https://pmc.ncbi.nlm.nih.gov/articles/PMC3229853/> |
|  | Inspiratory capacity | ↓ | <https://pubmed.ncbi.nlm.nih.gov/12841492/> |
|  | Peripheral obstruction | ­↑ | [https://doi.org/10.1016/j.rmed.2003.09.005; https://doi.org/10.4103/0970-2113.184875; https://doi.org/10.1016/S0140-6736(22)00470-6](https://doi.org/10.1016/j.rmed.2003.09.005) |
|  | Reactance area | ­↑ | [https://doi.org/10.4103/0970-2113.184875; https://doi.org/10.1016/S0140-6736(22)00470-6](https://doi.org/10.4103/0970-2113.184875) |
|  | Residual volume | ­↑ | <https://pmc.ncbi.nlm.nih.gov/articles/PMC3229853/> |
|  | Tidal volume (acute) | ­↑ | https://doi.org/10.1042/cs0610085; https://doi.org/10.1152/jappl.1985.58.6.1975 |
|  | Total lung capacity | ↓ | <https://pmc.ncbi.nlm.nih.gov/articles/PMC3229853/> |
|  | Vital capacity | ↓ | <https://pubmed.ncbi.nlm.nih.gov/12841492/> |
| Metabolic syndrome | Apolipoprotein A1 | ↓ | [https://doi.org/10.3109/08958378.2015.1013227; https://doi.org/10.1161/CIRCULATIONAHA.104.482570](https://doi.org/10.3109/08958378.2015.1013227) |
|  | Apolipoprotein B | ­↑ | <https://doi.org/10.1161/CIRCULATIONAHA.104.482570> |
|  | Blood glucose | ­↑ | https://doi.org/10.1093/ntr/ntx273; https://doi.org/10.1016/j.jacl.2009.10.008 |
|  | Hemoglobin glycosylated | ­↑ | https://doi.org/10.1093/ntr/ntx273; https://doi.org/10.3109/08958378.2015.1013227; https://doi.org/10.1080/1354750X.2017.1419284 |
|  | High-density lipoprotein cholesterol | ↓ | https://doi.org/10.1093/ntr/ntx273; https://doi.org/10.1080/1354750X.2017.1419284; https://doi.org/10.1016/j.jacl.2009.10.008 |
|  | Low-density lipoprotein cholesterol | ­↑ | https://doi.org/10.1093/ntr/ntx273; https://doi.org/10.1080/1354750X.2017.1419284; https://doi.org/10.1016/j.jacl.2009.10.008 |
|  | Total cholesterol | ­↑ | https://doi.org/10.1093/ntr/ntx273; https://doi.org/10.1016/j.jacl.2009.10.008 |
|  | Triglycerides | ­↑ | https://doi.org/10.1093/ntr/ntx273; https://doi.org/10.1016/j.jacl.2009.10.008 |
| Oral health | Bleeding on probing | ­↑ | <https://doi.org/10.2196/15350> |
|  | Clinical Attachment Loss | ­↑ | <https://doi.org/10.2196/15350> |
|  | Periodontal pocket depth | ­↑ | <https://doi.org/10.2196/15350> |
|  | Gingival inflammation | ­↑ | <https://doi.org/10.2196/15350> |
|  | Olsenella uli | ­↑ | [https://doi.org/10.1177/0022034515590581; https://doi.org/10.1186/s12941-022-00499-2](https://doi.org/10.1177/0022034515590581) |
|  | Plaque control record | ­↑ | <https://doi.org/10.2196/15350> |
|  | Porphyromonas gingivalis | ­↑ | https://doi.org/10.1111/omi.12273; https://doi.org/10.1016/j.disamonth.2011.03.008 |
|  | Pseudoramibacter alactolyticus | ­↑ | [https://doi.org/10.1177/0022034515590581; https://doi.org/10.1038/ismej.2014.114](https://doi.org/10.1177/0022034515590581) |
|  | Tannerella forsythia | ­↑ | [https://doi.org/10.1038/s41598-020-80937-3; https://doi.org/10.3389/froh.2021.751099](https://doi.org/10.1038/s41598-020-80937-3) |
|  | Tooth mobility | ­↑ | <https://doi.org/10.2196/15350> |
|  | Treponema denticola | ­↑ | [https://doi.org/10.1038/s41598-020-80937-3; https://doi.org/10.3389/froh.2021.751099](https://doi.org/10.1038/s41598-020-80937-3) |
| Oxidative stress | 8-epi-prostaglandin F2alpha | ­↑ | [https://doi.org/10.1093/ntr/ntx273; https://doi.org/10.1080/1354750X.2017.1419284](https://doi.org/10.1093/ntr/ntx273) |
|  | 8-hydroxy-2'-deoxyguanosine | ­↑ | https://doi.org/10.1080/10590500902885684; https://doi.org/10.1089/ars.2015.6508; https://doi.org/10.1093/carcin/18.9.1763; https://doi.org/10.3390/ijerph6020445 |
|  | 8-iso-prostaglandin F2alpha | ­↑ | [https://doi.org/10.1093/ntr/ntx273; https://doi.org/10.1080/1354750X.2017.1419284](https://doi.org/10.1093/ntr/ntx273) |
|  | H2O2 breakdown activity | ↓ | <https://doi.org/10.3390/antiox11091829> |
|  | H2O2 production | ­↑ | <https://doi.org/10.3390/antiox11091829> |
|  | Malondialdehyde | ­↑ | <https://doi.org/10.7759/cureus.60629> |
|  | Myeloperoxidase | ­↑ | [https://doi.org/10.1080/1354750X.2017.1419284; https://doi.org/10.1161/01.ATV.0000163262.83456.6d](https://doi.org/10.1080/1354750X.2017.1419284) |
|  | Protein carbonyls | ­↑ | [https://doi.org/10.1089/ars.2009.2887; https://doi.org/10.1016/j.cbi.2024.111008](https://doi.org/10.1089/ars.2009.2887) |
|  | Soluble Nox2-derived peptide | ­↑ | <https://doi.org/10.1016/j.chest.2016.04.012> |
|  | Total anti-oxidant capacity | ↓ | https://doi.org/10.5114/pja.2022.116285; https://doi.org/10.1186/1475-2891-6-39 |
|  | Vitamin E | ↓ | <https://doi.org/10.2147/CIA.S158513> |
| Platelet function & activation | 11-dehydrothromboxane B2 | ­↑ | [https://doi.org/10.3109/08958378.2015.1013227; https://doi.org/10.1080/1354750X.2017.1419284](https://doi.org/10.3109/08958378.2015.1013227) |
|  | Atheroma-chip, area under the curve | ­↑ | https://doi.org/10.1093/ntr/ntx273; https://doi.org/10.1007/s12012-023-09802-9 |
|  | Atheroma-chip, time to reach 10 kPa | ↓ | https://doi.org/10.1093/ntr/ntx273; https://doi.org/10.1007/s12012-023-09802-9 |
|  | Atheroma-chip, time to reach occlusion pressure | ↓ | https://doi.org/10.1093/ntr/ntx273; https://doi.org/10.1007/s12012-023-09802-9 |
|  | Fibrinogen | ­↑ | https://doi.org/10.1093/ntr/ntx273; https://doi.org/10.1080/1354750X.2017.1419284 |
|  | Platelet count | ­↑ | https://doi.org/10.1093/ntr/ntx273; https://doi.org/10.3109/08958378.2015.1013227 |
|  | Platelet-chip, area under the curve | ­↑ | https://doi.org/10.1093/ntr/ntx273; https://doi.org/10.1007/s12012-023-09802-9 |
|  | Platelet-chip, time to reach 10 kPa | ↓ | https://doi.org/10.1093/ntr/ntx273; https://doi.org/10.1007/s12012-023-09802-9 |
|  | Platelet-chip, time to reach occlusion pressure | ↓ | https://doi.org/10.1093/ntr/ntx273; https://doi.org/10.1007/s12012-023-09802-9 |
|  | Platelet-derived extracellular vesicles | ­↑ | https://doi.org/10.1111/jch.14479; https://doi.org/10.1164/rccm.201012-2061OC |
|  | P-selectin expressing extracellular vesicles | ­↑ | https://doi.org/10.1093/ntr/ntx273 |
|  | Soluble CD40 ligand | ­↑ | [https://doi.org/10.1161/CIRCULATIONAHA.104.482570; https://doi.org/10.1152/ajplung.00074.2015](https://doi.org/10.1161/CIRCULATIONAHA.104.482570) |
|  | Soluble P-selectin | ­↑ | https://doi.org/10.1093/ntr/ntx273; https://doi.org/10.3109/08958378.2015.1013227; https://doi.org/10.1080/1354750X.2017.1419284 |

# Supplementary Figure 1. PRISMA flow diagram for study selection. Abbreviations: HTP = heated tobacco; BoPH = biomarkers of potential harm; AE = adverse events.


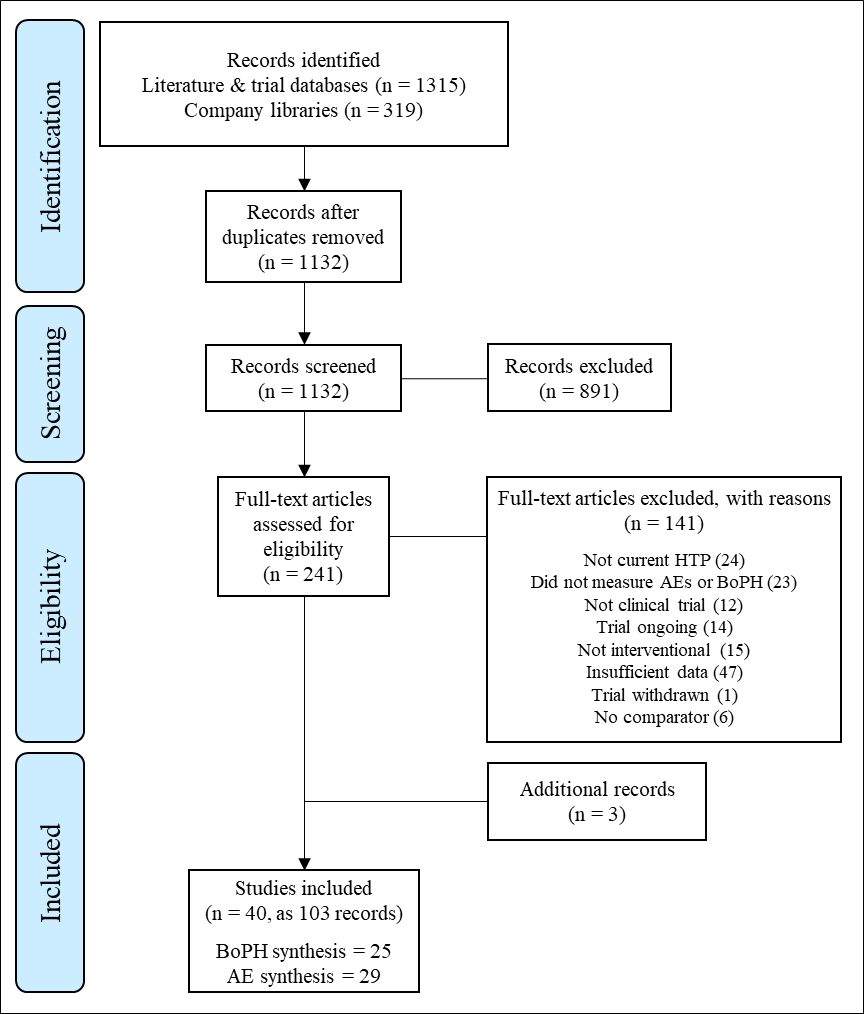


# Supplementary Table 2. Risk of bias judgements and support for judgements for each included study.

| **ISRCTN13439529** | | | | |  |
| --- | --- | --- | --- | --- | --- |
| **Bias** | **Authors’ judgements** | | **Support for judgement** | |  |
| Random sequence generation | Low | | "The order of use will be assigned by a pre-defined computer-generated randomisation schedule” | |  |
| Allocation concealment | Unclear | | "In ascending order of subject number, enrolled participants were assigned to receive the four study products in accordance with the pre-defined randomisation sequences, with an equal proportion of participants in each sequence" | |  |
| Blinding of participants and personnel | High | | "open-label" (Trial Reg & Poster). One active (NRT) and one non-active (CC) comparator. | |  |
| Blinding of outcome assessment | Low | | "open-label" (Trial Reg & Poster). Primary outcomes were objectively measured. | |  |
| Incomplete outcome data | Low | | Attrition 0% in all study groups. Exclusion: 23% of subjects excluded from analysis, "Seven subjects were excluded from the PK analysis population due to major protocol deviations (washout problem)" | |  |
| Selective reporting | Low | | All outcomes listed in the study protocol were fully reported on in at least one literature source | |  |
| **ISRCTN14301360/ UMIN000024988** | | | | |  |
| **Bias** | **Authors’ judgements** | | **Support for judgement** | |  |
| Random sequence generation | Low | | "The randomisation will be performed by Covance” | |  |
| Allocation concealment | Low | | "The randomisation will be performed by Covance and the clinics will enrol the participants and assign them to interventions" | |  |
| Blinding of participants and personnel | High | | "open-label" (Trial Reg & JA protocol). One active (Cess) and one non-active (CC) comparator. | |  |
| Blinding of outcome assessment | Low | | "open-label". All primary outcomes objectively measured. | |  |
| Incomplete outcome data | Low | | Overall attrition = 1.1%. No subjects who completed the study were excluded from the primary analyses. | |  |
| Selective reporting | High | | There were several outcomes listed in the protocol, namely biomarkers of effect and pharmacokinetic measures, that were not reported on. | |  |
| **ISRCTN80651909** | | | | |  |
| **Bias** | **Authors’ judgements** | | **Support for judgement** | |  |
| Random sequence generation | Low | | "The randomization will be computer-generated using SAS Version 9.3" | |  |
| Allocation concealment | Low | | “A randomisation scheme was provided for the clinical site to recruit 30 participants for each arm, giving a total of 150 participants” | |  |
| Blinding of participants and personnel | High | | "open-label" (Trial Reg & JA). Two active (Cess & EC) and one non-active (CC) comparator. | |  |
| Blinding of outcome assessment | Low | | "open-label". All primary outcomes objectively measured. | |  |
| Incomplete outcome data | Low | | Attrition: Glo=6.67% CC=0% EC=6.67% Cess=0% HTP=3.45%, overall=3.38%. Exclusion: Glo=6.67% CC=0% EC=6.67% Cess=0% HTP=N/A, overall=3.34%. | |  |
| Selective reporting | High | | No data reported for an entire study arm (C: "switching to a non-BAT commercial product"). No quantitative data reported for two biomarker of effect outcomes (WBC count & 8-epi-PGF2α Type III). No data reported for pharmacokinetic outcomes measured | |  |
| **ISRCTN81075760** | | | | |  |
| **Bias** | **Authors’ judgements** | | **Support for judgement** | |  |
| Random sequence generation | Low | | "randomised using blocks of computer-generated random number sequences" | |  |
| Allocation concealment | Unclear | | No information provided. | |  |
| Blinding of participants and personnel | High | | "This study will not be blinded" (Protocol supplementary file, pg 26)  Cigarette and non-smoker arms were non-active and subjects in the cessation arm received additional levels of support and products to aid smoking abstinence. | |  |
| Blinding of outcome assessment | Low | | "This study will not be blinded". All primary outcomes objectively measured. | |  |
| Incomplete outcome data | Unclear | | Number of participants randomised/enrolled, withdrawn and included in analyses vary between the 90-day, 180-day and 360-day reports. Entire arm (Group C) also removed. It is unclear when this group was removed from the study, why or how many participants were in the group. | |  |
| Selective reporting | High | | Several outcomes listed in the trial registration and protocol have not been reported on in any publications, including one of the primary endpoints, Augmentation index. | |  |
| **Dalrymple, 2022** | | | | |  |
| **Bias** | **Authors’ judgements** | | **Support for judgement** | |  |
| Random sequence generation | N/A | | Cochrane RoB tools designed to assess trials in which the unit of randomisation is people, rather than multiple sites on one individual, selection bias cannot be fairly assessed using this tool on this study. | |  |
| Allocation concealment | N/A | |  | |  |
| Blinding of participants and personnel | Unclear | | There is insufficient information provided in the text regarding blinding. One active (EC) and one non-active (CC) comparator. | |  |
| Blinding of outcome assessment | Low | | No information is provided in the text regarding blinding, but all primary outcomes objectively measured. | |  |
| Incomplete outcome data | Low | | All participants completed the study and none withdrew. | |  |
| Selective reporting | Unclear | | There was not trial registration or a priori protocol. | |  |
| **UMIN000017297** | | | | |  |
| **Bias** | **Authors’ judgements** | | **Support for judgement** | |  |
| Random sequence generation | Unclear | | Beyond stating the study was 'randomised', no further information provided. | |  |
| Allocation concealment | Unclear | | No information provided. | |  |
| Blinding of participants and personnel | High | | "Blinding: Open -no one is blinded" (trial reg). No active comparator (CC) | |  |
| Blinding of outcome assessment | Low | | "Blinding: Open-no one is blinded". All primary outcomes were objectively measured. | |  |
| Incomplete outcome data | Low | | All subjects randomised completed the study and were included in the analyses. | |  |
| Selective reporting | Low | | 3 safety profile parameters were not reported, but adverse events data were reported. All other outcomes listed in the methods and on the trial registration are reported on in at least one literature source. | |  |
| **UMIN000025777** | | | | |  |
| **Bias** | **Authors’ judgements** | | **Support for judgement** | |  |
| Random sequence generation | Unclear | | Beyond stating the study was 'randomised', no further information provided. | |  |
| Allocation concealment | Unclear | | No information provided. | |  |
| Blinding of participants and personnel | High | | "Blinding: Open -no one is blinded" (trial reg). No active comparator (CC) | |  |
| Blinding of outcome assessment | Low | | "Blinding: Open-no one is blinded". All primary outcomes were objectively measured. | |  |
| Incomplete outcome data | Low | | All subjects randomised completed the study and were included in the analyses. | |  |
| Selective reporting | Low | | 3 safety profile parameters were not reported, but adverse events data were reported. All other outcomes listed in the methods and on the trial registration are reported on in at least one literature source. | |  |
| **UMIN000041539** | | | | | |
| **Bias** | | **Authors’ judgements** | | **Support for judgement** | |
| Random sequence generation | | Low | | "Subjects were randomly assigned to one of six study groups using the electric data capture system and the investigators and site staffs were blinded to the randomization scheme." | |
| Allocation concealment | | Low | | "Subjects were randomly assigned to one of six study groups using the electric data capture system and the investigators and site staffs were blinded to the randomization scheme." | |
| Blinding of participants and personnel | | High | | "Open -no one is blinded". Included non-active comparator (cigarettes). | |
| Blinding of outcome assessment | | Low | | "Open -no one is blinded". All primary outcomes objectively measured. | |
| Incomplete outcome data | | Low | | Attrition: IT2.0a= 0% DT2.2a= 6.25% THP= 12.5% THS= 6.25% SS= 0% CC= 0%, overall= 4.3%. All completed participants included in analyses. | |
| Selective reporting | | Low | | All outcomes listed in the study protocol were fully reported on in at least one literature source | |
| **NCT02641587** | | | | |  |
| **Bias** | **Authors’ judgements** | | **Support for judgement** | |  |
| Random sequence generation | Low | | "subjects will be randomized using an interactive web and voice response system (IxRS)" | |  |
| Allocation concealment | Low | | "subjects will be randomized using an interactive web and voice response system (IxRS)" | |  |
| Blinding of participants and personnel | High | | "Masking: None (Open Label)" Trial reg. No active comparator (CC) | |  |
| Blinding of outcome assessment | Low | | "Masking: None (Open Label)". All primary outcomes objectively measured. | |  |
| Incomplete outcome data | Low | | Attrition: IQOS=5% CC=2.5%, overall=4.17%. Exclusion: IQOS=3.75% CC=12.5%, overall=6.6%. | |  |
| Selective reporting | High | | "Samples for 4-HNE analysis have been collected but will not be analyzed due to the failure to develop a selective and quantitative assay."  QSU, Intent to Use of CHTP 1.2, Prochaska “Stage of Change” Questionnaire, MCEQ, and pre- and post-bronchodilator FVC, FEV1/FVC, FEF 25-75 were not reported in any literature sources. | |  |
| **NCT01959607** | | | | |  |
| **Bias** | **Authors’ judgements** | | **Support for judgement** | |  |
| Random sequence generation | Low | | "Randomization to each product exposure sequence was done through an Interactive Telephone and Web Response System." | |  |
| Allocation concealment | Low | | "Randomization to each product exposure sequence was done through an Interactive Telephone and Web Response System." | |  |
| Blinding of participants and personnel | High | | "This was an open-label study; therefore the subjects and investigators were unblinded to subjects' sequence." No supportive products or behavioural support were provided to any participants during the trial, but in one arm the comparator intervention was non-active (cigarettes). | |  |
| Blinding of outcome assessment | Low | | "This was an open-label study". All primary outcomes objectively measured. | |  |
| Incomplete outcome data | Low | | Attrition: IQOS-CC=5%, IQOS-NRT=0%. No participants who completed the trial were excluded from the analyses. | |  |
| Selective reporting | Low | | All outcomes reported in at least one literature source. | |  |
| **NCT02503254** | | | | |  |
| **Bias** | **Authors’ judgements** | | **Support for judgement** | |  |
| Random sequence generation | Low | | "subjects were randomized by an interactive web and voice response system” | |  |
| Allocation concealment | Low | | "subjects were randomized by an interactive web and voice response system” | |  |
| Blinding of participants and personnel | High | | "Masking: None (Open Label)" (Trial reg). No active comparator (CC) | |  |
| Blinding of outcome assessment | Low | | "Masking: None (Open Label)". All primary outcomes objectively measured. | |  |
| Incomplete outcome data | Low | | Attrition and exclusion both 0%. | |  |
| Selective reporting | High | | Several outcomes listed in the study protocol were not reported on in the main results article. Only one was reported on in a poster instead. | |  |
| **NCT01967719** | | | | |  |
| **Bias** | **Authors’ judgements** | | **Support for judgement** | |  |
| Random sequence generation | Low | | "Randomization to each product exposure sequence was done through an Interactive Telephone and Web Response System" | |  |
| Allocation concealment | Low | | "Randomization to each product exposure sequence was done through an Interactive Telephone and Web Response System" | |  |
| Blinding of participants and personnel | High | | "Masking: None (Open Label)" (Trial Reg). One active (NRT) and one non-active (CC) comparator. | |  |
| Blinding of outcome assessment | Low | | "Masking: None (Open Label)". All primary outcomes objectively measured. | |  |
| Incomplete outcome data | Low | | Attrition: IQOS/CC=4.55% IQOS/NRT=0%, overall=3.23%. No subjects who completed the study were excluded from the analysis. | |  |
| Selective reporting | Low | | All outcomes reported in at least one literature source. | |  |
| **NCT01989156** | | | | |  |
| **Bias** | **Authors’ judgements** | | **Support for judgement** | |  |
| Random sequence generation | Low | | "randomization was done through the Interactive Web and Voice Response System (IWRS)" | |  |
| Allocation concealment | Low | | "randomization was done through the Interactive Web and Voice Response System (IWRS)" | |  |
| Blinding of participants and personnel | High | | "This is an open-label study; therefore, the subjects and Investigators will be unblinded to subject’s arm" (Study protocol) The level of support given to each arm differed: "All subjects in the SA arm will receive smoking cessation counselling and will be able to use nicotine replacement therapy (NRT) if considered necessary by the Investigator or requested by the subject" | |  |
| Blinding of outcome assessment | Low | | "This is an open-label study”. All primary outcomes objectively measured. | |  |
| Incomplete outcome data | Low | | Attrition: IQOS=9%, CC=15%, SA=21%. Although the primary analysis used per-protocol populations, results data for the full analysis set were also provided in the clinical study report. | |  |
| Selective reporting | Low | | All outcomes reported in at least one literature source. | |  |
| **NCT01970982** | | | | |  |
| **Bias** | **Authors’ judgements** | | **Support for judgement** | |  |
| Random sequence generation | Low | | "randomization was performed through an Interactive Web and Voice Response System" | |  |
| Allocation concealment | Low | | "randomization was performed through an Interactive Web and Voice Response System" | |  |
| Blinding of participants and personnel | High | | "Masking: None (Open Label)" (Trial Reg). One active (Cess) and one non-active (CC) comparator. | |  |
| Blinding of outcome assessment | Low | | "Masking: None (Open Label)". All primary outcomes objectively measured. | |  |
| Incomplete outcome data | Low | | Attrition: IQOS=0% CC=0% Cess=5%, overall=1.25%. All subjects who completed the study were included in the analysis. | |  |
| Selective reporting | Low | | All outcomes reported in at least one literature source. | |  |
| **NCT01959932** | | | | |  |
| **Bias** | **Authors’ judgements** | | **Support for judgement** | |  |
| Random sequence generation | Low | | "randomization was done through an Interactive Web and Voice Response System" | |  |
| Allocation concealment | Low | | "randomization was done through an Interactive Web and Voice Response System" | |  |
| Blinding of participants and personnel | High | | "Masking: None (Open Label)" (Trial Reg). One active (Cess) and one non-active (CC) comparator. | |  |
| Blinding of outcome assessment | Low | | "Masking: None (Open Label)". All primary outcomes objectively measured. | |  |
| Incomplete outcome data | Low | | Attrition: IQOS=1.25% CC=0% Cess=0%, overall=0.62%. All subjects who completed the study were included in the analysis. | |  |
| Selective reporting | Low | | All outcomes reported in at least one literature source. | |  |
| **NCT01780714** | | | | |  |
| **Bias** | **Authors’ judgements** | | **Support for judgement** | |  |
| Random sequence generation | Unclear | | Beyond stating the study was 'randomised', no further information provided. | |  |
| Allocation concealment | Unclear | | No information provided. | |  |
| Blinding of participants and personnel | High | | "Masking: None (Open Label)" (Trial reg). No active comparator (CC) | |  |
| Blinding of outcome assessment | Low | | "Masking: None (Open Label)". All primary outcomes objectively measured. | |  |
| Incomplete outcome data | Low | | All participants randomised completed the trial and no participants were excluded from the analysis. | |  |
| Selective reporting | High | | Data for 4 outcomes listed in the protocol (Cytochrome P450 2A6 activity, Questionnaire of Smoking Urges, Minnesota Nicotine Withdrawal Scale, Respiratory symptoms) were not reported. | |  |
| **NCT02396381** |  | |  | |  |
| **Bias** | **Authors’ judgements** | | **Support for judgement** | |  |
| Random sequence generation | Low | | "Randomization was done through the interactive voice and web response system (IXRS)" | |  |
| Allocation concealment | Low | | "Randomization was done through the interactive voice and web response system (IXRS)" | |  |
| Blinding of participants and personnel | High | | "Masking: None (Open Label)" (Trial reg). No active comparator (CC) | |  |
| Blinding of outcome assessment | Low | | "Masking: None (Open Label)". All primary outcomes objectively measured | |  |
| Incomplete outcome data | Low | | Attrition: IQOS=15.16% CC=10.69%, overall=2.91%. Although not the main analysis population, full analysis set (as randomised) results data were also presented in the published literature. | |  |
| Selective reporting | Low | | All outcomes reported on in at least one literature source. | |  |
| **NCT01970995** |  | |  | |  |
| **Bias** | **Authors’ judgements** | | **Support for judgement** | |  |
| Random sequence generation | Low | | "randomization was performed through the Interactive Web and Voice Response System" | |  |
| Allocation concealment | Low | | "randomization was performed through the Interactive Web and Voice Response System" | |  |
| Blinding of participants and personnel | High | | "Masking: None (Open Label)" (Trial Reg). One active (Cess) and one non-active (CC) comparator. | |  |
| Blinding of outcome assessment | Low | | "Masking: None (Open Label)". All primary outcomes objectively measured. | |  |
| Incomplete outcome data | Low | | Attrition: IQOS=2.56% CC=2.38% Cess=5%, overall=3.12%. Exclusion: IQOS=10.26% CC=2.4% Cess=7.5%, overall=7.5%. | |  |
| Selective reporting | Low | | All outcomes reported in at least one literature source. | |  |
| **NCT02466412** | | | | |  |
| **Bias** | **Authors’ judgements** | | **Support for judgement** | |  |
| Random sequence generation | Low | | "Randomization to product exposure sequence will be done through IxRS" | |  |
| Allocation concealment | Low | | "Randomization to product exposure sequence will be done through IxRS" | |  |
| Blinding of participants and personnel | High | | "Masking: None (Open Label)" (Trial reg). No active comparator (CC) | |  |
| Blinding of outcome assessment | Low | | "Masking: None (Open Label)". All primary outcomes were objectively measured. | |  |
| Incomplete outcome data | Low | | Attrition was 0%. Exclusion: mCHTP-mCC=4.16% mCC-mCHTP=0%, overall=2.1% | |  |
| Selective reporting | High | | Only results data for the two primary outcomes have thus far been published. | |  |
| **NCT02649556** | | | | |  |
| **Bias** | **Authors’ judgements** | | **Support for judgement** | |  |
| Random sequence generation | Low | | "Randomization was done during the original study at V4 through the interactive voice and web response system (IXRS)." | |  |
| Allocation concealment | Low | | "Randomization was done during the original study at V4 through the interactive voice and web response system (IXRS)." | |  |
| Blinding of participants and personnel | High | | "Masking: None (Open Label)" (Trial reg). No active comparator (CC) | |  |
| Blinding of outcome assessment | Low | | "Masking: None (Open Label)". All primary outcomes objectively measured. | |  |
| Incomplete outcome data | High | | Attrition rates in all arms were <50% (IQOS=8%, CC=9%) and differed by <20%. However results data reported are based on participant product use not randomisation. True ITT or FAS sets were not provided | |  |
| Selective reporting | High | | Several outcomes listed in the study protocol have not been reported on in any publications. | |  |
| Other | High | | Only reported data grouped by participant product use not randomisation. | |  |
| **NCT01967732** | | | | |  |
| **Bias** | **Authors’ judgements** | | **Support for judgement** | |  |
| Random sequence generation | Low | | "Randomization to product exposure sequence was performed through an Interactive Telephone and Web Response System" | |  |
| Allocation concealment | Low | | "Randomization to product exposure sequence was performed through an Interactive Telephone and Web Response System" | |  |
| Blinding of participants and personnel | High | | "Masking: None (Open Label)" (Trial Reg). One active (NRT) and one non-active (CC) comparator. | |  |
| Blinding of outcome assessment | Low | | "Masking: None (Open Label)". All primary outcomes objectively measured. | |  |
| Incomplete outcome data | Low | | Attrition: IQOS/CC=4.55% IQOS/NRT=5.56%, overall=4.84%. Exclusion: IQOS/CC=6.81% IQOS/NRT=5.5%, overall=6.45%. | |  |
| Selective reporting | Low | | All outcomes reported in at least one literature source. | |  |
| **NCT01780688** | | | | |  |
| **Bias** | **Authors’ judgements** | | **Support for judgement** | |  |
| Random sequence generation | Low | | "Randomization was performed using an Interactive Web Response System" | |  |
| Allocation concealment | Low | | "Randomization was performed using an Interactive Web Response System" | |  |
| Blinding of participants and personnel | High | | "Masking: None (Open Label)" (Trial reg). No active comparator (CC) | |  |
| Blinding of outcome assessment | Low | | "Masking: None (Open Label)". All primary outcomes objectively measured. | |  |
| Incomplete outcome data | Low | | All participants randomised completed the trial and no participants were excluded from the analysis. | |  |
| Selective reporting | Low | | All outcomes reported on in at least one literature source. | |  |
| **NCT01967706** | | | | |  |
| **Bias** | **Authors’ judgements** | | **Support for judgement** | |  |
| Random sequence generation | Low | | "Randomization to product exposure sequence was done through an Interactive Telephone and Web Response System" | |  |
| Allocation concealment | Low | | "Randomization to product exposure sequence was done through an Interactive Telephone and Web Response System" | |  |
| Blinding of participants and personnel | High | | "Masking: None (Open Label)" (Trial reg). 1 active (NRT) and 1 non-active (CC) comparator | |  |
| Blinding of outcome assessment | Low | | "Masking: None (Open Label)". All primary outcomes objectively measured. | |  |
| Incomplete outcome data | Low | | Attrition: IQOS-CC=2.27% IQOS-NRT=0%, overall=1.61%. No subjects who completed the study were excluded from the analysis. | |  |
| Selective reporting | Low | | All outcomes reported on in at least one literature source. | |  |
| **NCT03364751** | | | | |  |
| **Bias** | **Authors’ judgements** | | **Support for judgement** | |  |
| Random sequence generation | Low | | "Randomization will be done through the Interactive Web and Voice Response System (IXRS)” | |  |
| Allocation concealment | Low | | "Randomization will be done through the Interactive Web and Voice Response System (IXRS)” | |  |
| Blinding of participants and personnel | High | | "Masking: Single (Investigator)" (trial reg). No active comparator (CC) | |  |
| Blinding of outcome assessment | Low | | "Masking: Single (Investigator)". Primary outcome objectively assessed. | |  |
| Incomplete outcome data | Low | | Attrition: IQOS=1.15% CC=1.18%, overall=1.16%. Exclusion: IQOS=19.54% CC=1.18%, overall=1.74%. | |  |
| Selective reporting | High | | Results data from "Full transcriptomics profile assessment of buccal swabs derived from the right and left buccal mucosa (3 and 6 months)" has not been reported | |  |
| Other | High | | Only reported data grouped by participant product use not randomisation. | |  |
| **DRKS00012919** | | | | |  |
| **Bias** | **Authors’ judgements** | | **Support for judgement** | |  |
| Random sequence generation | Unclear | | Beyond stating the study was 'randomised', no further information provided. | |  |
| Allocation concealment | Unclear | | No information provided. | |  |
| Blinding of participants and personnel | High | | "partly double-blinded": Only two interventions/arms were blinded (EC+/EC-). Two active (EC+ & EC-) and one non-active (CC) comparator. | |  |
| Blinding of outcome assessment | Low | | Only the e-cigarette arms were blinded. All primary outcomes objectively measured. | |  |
| Incomplete outcome data | Unclear | | In the protocol the target for enrolment was 55. It is unclear whether 55 completed the study and only 20 were included in the analyses. | |  |
| Selective reporting | High | | In the trial registration, the authors state outcomes relating to "endothelial dysfunction and inflammatory markers" were measured. No specific measures were given and no relevant data were reported. | |  |
| **NCT03301129** | | | | |  |
| **Bias** | **Authors’ judgements** | | **Support for judgement** | |  |
| Random sequence generation | Low | | "The randomization list was computer generated" | |  |
| Allocation concealment | Unclear | | No information provided. | |  |
| Blinding of participants and personnel | Unclear | | Despite describing the trial as "Double" blinded on the trial registration, only "Investigator" and "Outcome Assessor" are noted as being masked, not participants. | |  |
| Blinding of outcome assessment | Low | | "Masking: Double (Investigator, Outcomes Assessor)". Primary outcomes were objectively measured | |  |
| Incomplete outcome data | Low | | The 30 subjects excluded were excluded pre-randomisation. No subjects who were randomised withdrew or were excluded from the final analysis population. | |  |
| Selective reporting | Low | | All outcomes reported on in at least one literature source. | |  |
| **NCT03435562** | | | | |  |
| **Bias** | **Authors’ judgements** | | **Support for judgement** | |  |
| Random sequence generation | Low | | "Order of the products used in each session will be assigned using Latin-square order procedure” | |  |
| Allocation concealment | Unclear | | No information provided. | |  |
| Blinding of participants and personnel | High | | Masking: None (Open Label) (trial reg). 1 active (EC) and 1 non-active (CC) comparators | |  |
| Blinding of outcome assessment | Low | | “Masking: None (Open Label)”. Primary outcome objectively measured. | |  |
| Incomplete outcome data | Low | | Overall attrition = 18.18%. All participants who completed the study were included in the analysis. | |  |
| Selective reporting | High | | Results data for heart rate and blood pressure have not been reported. | |  |
| **NCT03452124** | | | | |  |
| **Bias** | **Authors’ judgements** | | **Support for judgement** | |  |
| Random sequence generation | Low | | "Randomization was performed by an attending research nurse using a table of random numbers as reproduced from the online randomization software http://www.graphpad.com/quickcalcs/index.cfm" | |  |
| Allocation concealment | Unclear | | There is insufficient information provided to determine whether intervention allocation was concealed | |  |
| Blinding of participants and personnel | Unclear | | Trial registration states "Masking: Quadruple (Participant, Care Provider, Investigator, Outcomes Assessor)", but in the publication the only blinding described is in regard to outcome assessors. | |  |
| Blinding of outcome assessment | Low | | "examinations were executed by a single, blindedto-treatment and to values of measured biomarkers, operator". Outcomes were physiological measures. | |  |
| Incomplete outcome data | Low | | All participants completed the study and none withdrew. | |  |
| Selective reporting | High | | Not all outcomes measured were reported on. | |  |
| **Ioakeimidis, 2021** | | | | |  |
| **Bias** | **Authors’ judgements** | | **Support for judgement** | |  |
| Random sequence generation | Unclear | | Whether or how participants were randomised is unclear. | |  |
| Allocation concealment | Unclear | | How interventions were allocated is not described. | |  |
| Blinding of participants and personnel | Unclear | | No information is provided in the text regarding blinding. Non-active (CC) comparator. | |  |
| Blinding of outcome assessment | Low | | Outcomes were physiological measures. | |  |
| Incomplete outcome data | Unclear | | The authors state they "studied 22 current smokers" but it is unclear whether more than 22 were initially randomised or enrolled. | |  |
| Selective reporting | Unclear | | There was not trial registration or a priori protocol. | |  |
| **Lopez, 2016** | | | | |  |
| **Bias** | **Authors’ judgements** | | **Support for judgement** | |  |
| Random sequence generation | Low | | "Participants completed each of the three, Latin-square ordered, ∼2.5-h sessions" (JA) | |  |
| Allocation concealment | Unclear | | There is no information provided to determine the presence or level of intervention allocation concealment | |  |
| Blinding of participants and personnel | Unclear | | No information is provided in the text regarding blinding. One active (EC) and one non-active (CC) comparator. | |  |
| Blinding of outcome assessment | Low | | No information is provided in the text regarding blinding. Primary outcomes were objectively measured. | |  |
| Incomplete outcome data | Low | | Overall attrition = 37.5%. No subjects who completed the study were excluded form the analysis. | |  |
| Selective reporting | Unclear | | There was not trial registration or a priori protocol. | |  |
| **Yaman, 2021** | | | | |  |
| **Bias** | **Authors’ judgements** | | **Support for judgement** | |  |
| Random sequence generation | Unclear | | Participants were already IQOS users. It is unclear how participants were randomised to CC or HTP. | |  |
| Allocation concealment | Unclear | | Staff asked participants to use products, ie. They were aware. It is not clear if the order of interventions was randomised. | |  |
| Blinding of participants and personnel | Unclear | | No information is provided in the text regarding blinding. Non-active (CC) comparator. | |  |
| Blinding of outcome assessment | Low | | Outcomes were physiological measures. | |  |
| Incomplete outcome data | Low | | Reasons for withdrawal are clearly described. | |  |
| Selective reporting | Unclear | | There was not trial registration or a priori protocol. | |  |
| **NCT06093659** | | | | | |
| **Bias** | | **Authors’ judgements** | | **Support for judgement** | |
| Random sequence generation | | Unclear | | Participants "randomised” but no details of method. | |
| Allocation concealment | | Unclear | | Participants "randomised” but no details of method. | |
| Blinding of participants and personnel | | High | | "Masking: None (Open Label)" (Trial reg). No active comparator (CC). | |
| Blinding of outcome assessment | | Low | | "Masking: None (Open Label)" (Trial reg). However all primary outcomes were objectively measured. | |
| Incomplete outcome data | | Unclear | | Number of participants lost to attrition and/or excluded from analyses not reported. | |
| Selective reporting | | High | | Puff count not reported. | |
| **Yuki, 2023** | | | | | |
| **Bias** | | **Authors’ judgements** | | **Support for judgement** | |
| Random sequence generation | | Unclear | | Participants "randomised” but no details of method. | |
| Allocation concealment | | Unclear | | Participants "randomised” but no details of method. | |
| Blinding of participants and personnel | | Unclear | | There is insufficient information provided in the text regarding blinding. Two active (EC & SA) and one non-active (CC) comparator. | |
| Blinding of outcome assessment | | Low | | There is insufficient information provided in the text regarding blinding. Primary outcomes objectively measured. | |
| Incomplete outcome data | | High | | Attrition rates in all arms were <50% (highest 47.1% - Cohort H) but there was a 30.9% difference between the highest and lowest cohorts (Cohort F vs Cohort H). | |
| Selective reporting | | Unclear | | There was not trial registration or a priori protocol. | |
| **UMIN000045304** | | | | | |
| **Bias** | | **Authors’ judgements** | | **Support for judgement** | |
| Random sequence generation | | Low | | "The subjects were randomly assigned into the study groups by using the electronic data capture system, and the investigators and site staff were blinded to the randomization scheme." | |
| Allocation concealment | | Low | | "The subjects were randomly assigned into the study groups by using the electronic data capture system, and the investigators and site staff were blinded to the randomization scheme." | |
| Blinding of participants and personnel | | High | | "open-label". Both active and non-active comparators. | |
| Blinding of outcome assessment | | Low | | "open-label". However all primary outcomes were objectively measured. | |
| Incomplete outcome data | | Low | | Attrition 2% overall, and no participants excluded from analyses. | |
| Selective reporting | | Low | | All outcomes were reported on. | |
| **NCT05459857** | | | | | |
| **Bias** | | **Authors’ judgements** | | **Support for judgement** | |
| Random sequence generation | | Low | | Subjects who completed the study screening assessments were assigned a unique randomisation identification number. Subsequently, each subject, based on the identification number, were assigned to use the study products according to one of four product sequences, which were prepared by Celerion, Inc. | |
| Allocation concealment | | Low | | Subjects who completed the study screening assessments were assigned a unique randomisation identification number. Subsequently, each subject, based on the identification number, were assigned to use the study products according to one of four product sequences, which were prepared by Celerion, Inc. | |
| Blinding of participants and personnel | | High | | Masking: None (Open Label) (Trial reg). No active comparator (CC). | |
| Blinding of outcome assessment | | Low | | "Masking: None (Open Label)" (Trial reg). However all primary outcomes were objectively measured. | |
| Incomplete outcome data | | Low | | All participants randomised completed the trial and no participants were excluded from the analysis. | |
| Selective reporting | | Low | | All outcomes were reported on. | |
| **NCT05114863** | | | | | |
| **Bias** | | **Authors’ judgements** | | **Support for judgement** | |
| Random sequence generation | | Low | | The study statistician at the contract research organization generated the random allocation sequence and its implementation. | |
| Allocation concealment | | Low | | The study statistician at the contract research organization generated the random allocation sequence and its implementation. | |
| Blinding of participants and personnel | | High | | By necessity, this study was unblinded due to the different visual appearances of the study products. | |
| Blinding of outcome assessment | | Low | | "Masking: None (Open Label)" (Trial reg). Primary outcomes were objectively measured. | |
| Incomplete outcome data | | Low | | Attrition 8% overall, and no participants excluded from analyses. | |
| Selective reporting | | Low | | All outcomes were reported on. | |
| **DRKS00020446** | | | | | |
| **Bias** | | **Authors’ judgements** | | **Support for judgement** | |
| Random sequence generation | | Low | | Participants "randomised” but no details of method. | |
| Allocation concealment | | Unclear | | Participants "randomised” but no details of method. | |
| Blinding of participants and personnel | | Unclear | | No details provided regarding blinding. Both active and non-active comparators. | |
| Blinding of outcome assessment | | Low | | No details provided regarding blinding. However outcomes were objectively measured. | |
| Incomplete outcome data | | Unclear | | 17 participants included, but details of how many were recruited was not reported. | |
| Selective reporting | | Unclear | | The outcomes listed on the trial registration were not reported on in the publication. It appears the registration may have been for a different study. | |
| **ChiCTR2200065055** | | | | | |
| **Bias** | | **Authors’ judgements** | | **Support for judgement** | |
| Random sequence generation | | Low | | "Eligible participants were randomized through a computer-generated sequence utilizing a simple randomization algorithm in SAS version 9.4." | |
| Allocation concealment | | Low | | "Eligible participants were randomized through a computer-generated sequence utilizing a simple randomization algorithm in SAS version 9.4." | |
| Blinding of participants and personnel | | High | | "Given the open-label nature of the trial, both investigators and participants were cognizant of group assignments. However, the statistical analysts were blinded during data analysis." Non-active comparator (CC) | |
| Blinding of outcome assessment | | Low | | "open-label". However all primary outcomes were objectively measured. | |
| Incomplete outcome data | | Low | | Attrition rates in all arms were <50% (HTP=0%, CC=0%) and differed by <20%. All participants included in analyses | |
| Selective reporting | | Low | | All outcomes were reported on. | |
| **Lyytinen, 2024** | | | | | |
| **Bias** | | **Authors’ judgements** | | **Support for judgement** | |
| Random sequence generation | | Unclear | | Participants "randomised” but no details of method. | |
| Allocation concealment | | Unclear | | Participants "randomised” but no details of method. | |
| Blinding of participants and personnel | | Unclear | | No details provided regarding blinding. One active comparator (no exposure), but not similar intensity. | |
| Blinding of outcome assessment | | Low | | No details provided regarding blinding. However outcomes were objectively measured. | |
| Incomplete outcome data | | Low | | The authors note that due to a loss of data, they had to recruit an additional 8 individuals. All but one of the 32 (original 24 + 8) participants randomised completed the study (attrition 4%). "23 individuals completed the arterial stiffness analysis and 22 the T-TAS analysis" (exclusion <30%). | |
| Selective reporting | | Unclear | | There was not trial registration or a priori protocol. | |
|  | | | | | |
| **Bias** | | **Authors’ judgements** | | **Support for judgement** | |
| Random sequence generation | | Unclear | | Participants "randomised” but no details of method. | |
| Allocation concealment | | Unclear | | Participants "randomised” but no details of method. | |
| Blinding of participants and personnel | | Unclear | | "All statistical analyses were conducted by an investigator who was blinded to the group allocations." But otherwise no further details given regarding blinding. One active comparator (no exposure), but not similar intensity. | |
| Blinding of outcome assessment | | Low | | Insufficient details provided regarding blinding. However outcomes were objectively measured. | |
| Incomplete outcome data | | High | | 24 enrolled & randomised. 23 included in analyses, unclear if missing one was due to withdrawal or exclusion. A further participant was also excluded from analyses due to being an "outlier". | |
| Selective reporting | | Unclear | | The outcomes listed on the trial registration were not reported on in the publication. It appears the registration may have been for a different study. | |

# Supplementary Figure 2. Summary plot showing risk of bias judgements across studies.


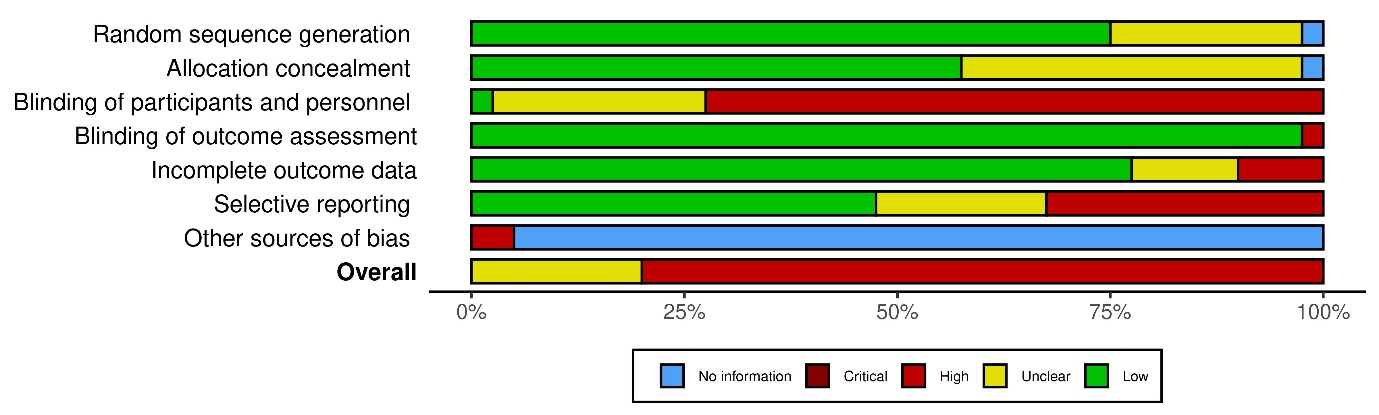


# Supplementary Figure 3. Risk of bias traffic light plot: review authors’ judgments about risk of bias items for each included study.

**
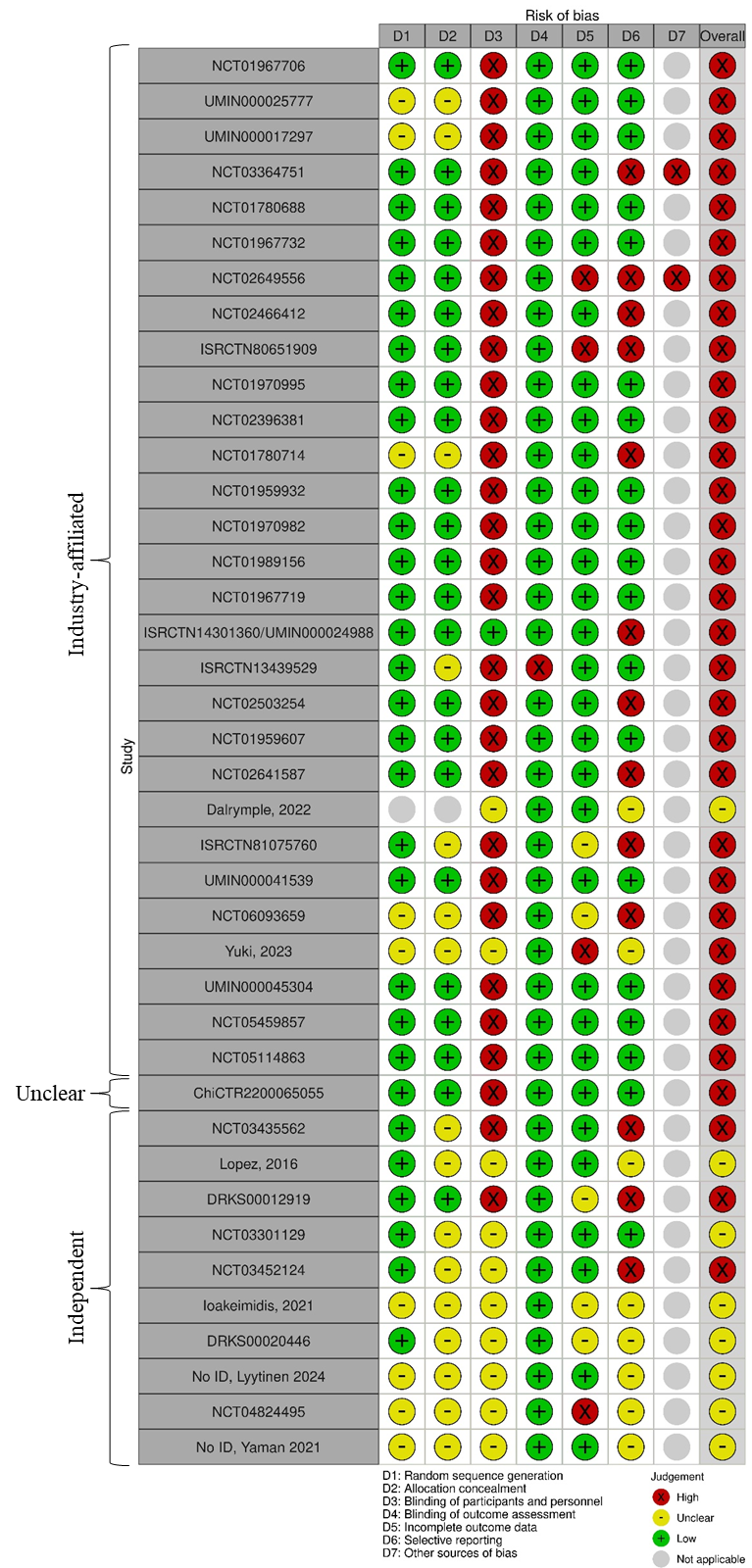
**

# Supplementary Figure 4. Direction of effect between baseline and last follow-up in HTP arms in confined studies.


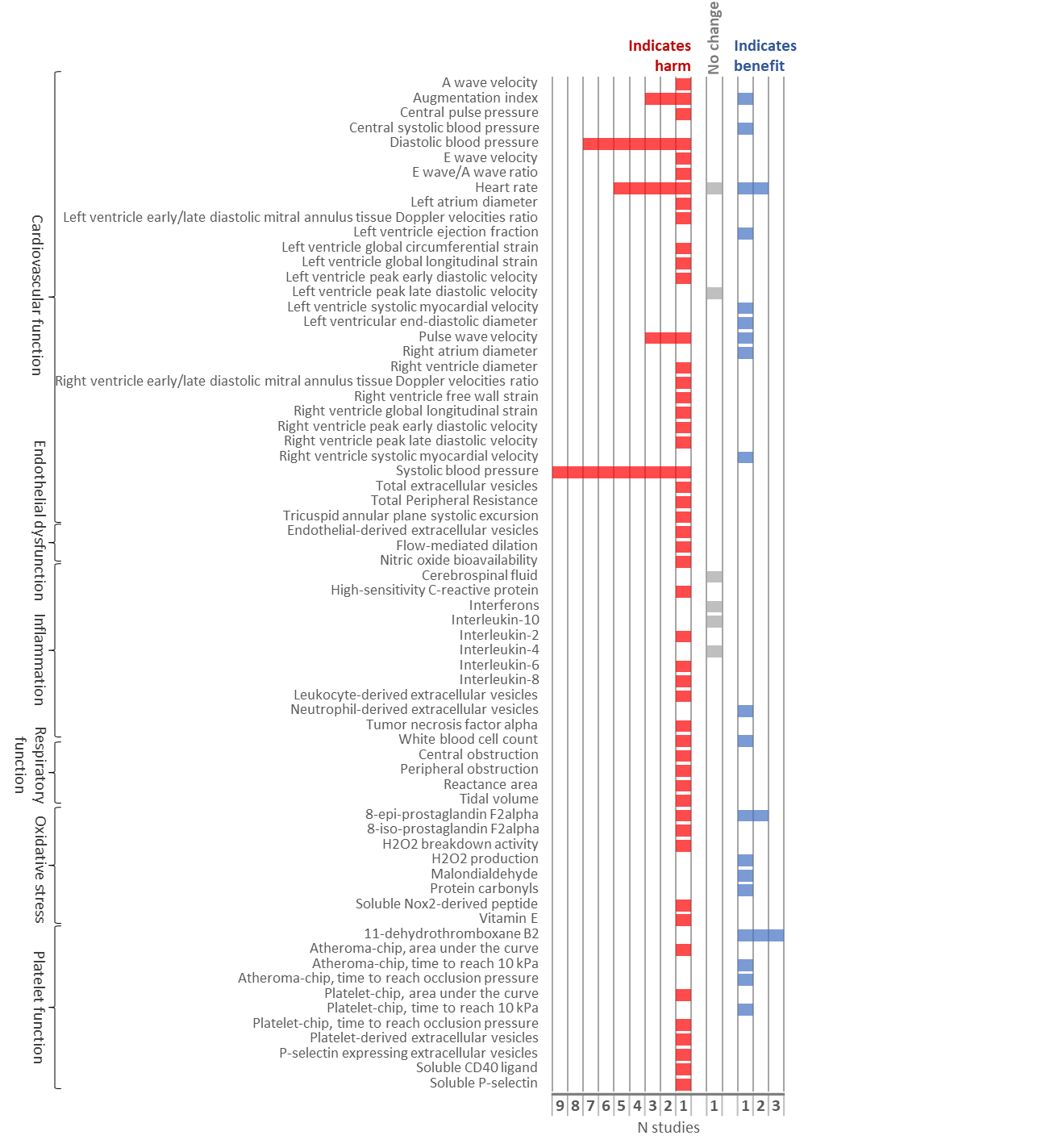


# Supplementary Figure 5. Direction of effect between baseline and last follow-up in HTP arms in ambulatory studies.

Abbreviations: BDR = bronchodilator response.


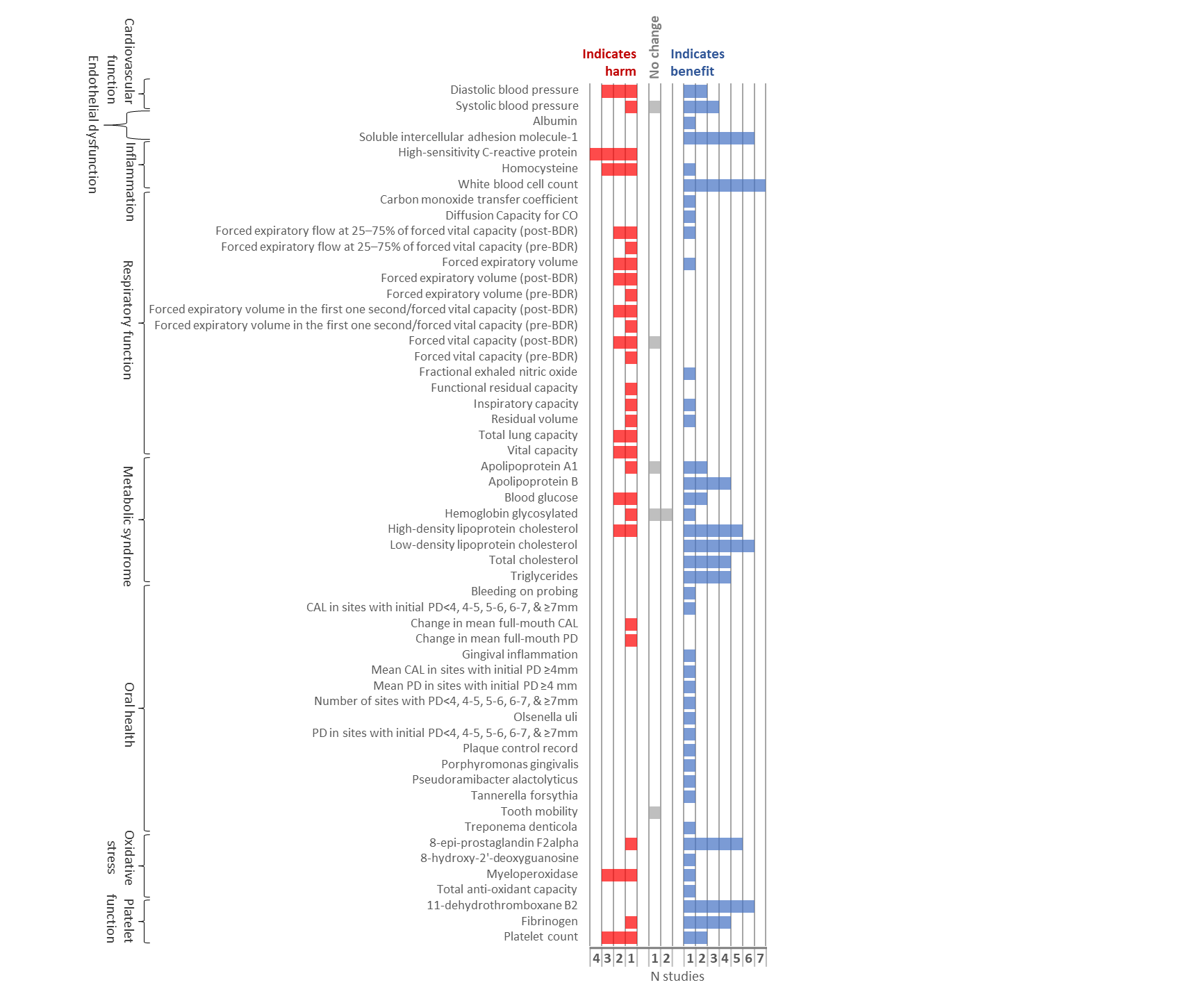


# Supplementary Table 3. Serious and non-serious adverse event data from crossover trials.

| Study | Extracted data |
| --- | --- |
| NCT01959607 | Format: n (%) subjects; [n] AEs  **THS-CC:**  AEs= 3 (13.6%) [3]  Mild AEs= 2 (9.1%) [2]  Moderate AEs= 1 (4.5%)[1]  Severe AEs= 0 (0%)  SAEs= 0 [0]  AEs related to IP= 0 [0]  AEs related to NRT= 0 [0]  AEs related to study procedures= 0 [0]  **CC-THS:**  AEs= 3 (13.6%) [5]  Mild AEs= 2 (9.1%) [4]  Moderate AEs= 1 (5.4%) [1]  Severe AEs= 0 [0]  SAEs= 0 [0]  AEs related to IP= 2 (9.1%) [2]  Dysphoria= 1 (4.5%)  Hepatic function abnormal= 1 (4.5%)  AEs related to NRT= 0 [0]  AEs related to study procedures= 1 (4.5%) [3]  **THS-NRT:**  AEs= 4 (44.4%) [5]  Mild AEs= 3 (33.3%) [4]  Moderate AEs= 1 (11.1%) [1]  Severe AEs= 0 [0]  SAEs= 0 [0]  AEs related to IP= 1 (11.1%) [1]  Dysphoria= 1 (11.1%)  AEs related to NRT= 0 [0]  AEs related to study procedures= 2 (2.2%) [2]  **NRT-THS:**  AEs= 1 (11.1%) [1]  Mild AEs= 1 (11.1%) [1]  Moderate AEs= 0 [0]  Severe AEs= 0 [0]  SAEs= 0 [0]  AEs related to IP= 0 [0]  AEs related to NRT= 0 [0]  AEs related to study procedures= 0 [0] |
| NCT01967719 | Format: n (%) subjects; [n] AEs  **THS2.2-CC:**  AEs= 7 (31.8%) [10]  SAEs= 0 [0]  Severe AEs= 0 [0]  AEs related to IP= 2 (9.1%) [3]  Headache= 1 (4.5%)  Vomiting= 1 (4.5%)  Nausea= 1 (4.5%)  AEs related to NNS= 1 (4.5%)  Sneezing= 1 (4.5%)  AEs related to study procedures= 2 (9.1%) [4]  **CC-THS2.2:**  AEs= 7 (31.8%) [9]  SAEs= 0 [0]  Severe AEs= 0 [0]  AEs related to IP= 1 (4.5%) [1]  Vomiting= 1 (4.5%)  AEs related to study procedures= 2 (9.1%) [2]  **THS2.2-NNS:**  AEs= 3 (3.33%) [7]  SAEs= 0 [0]  Severe AEs= 1 (11.1%) [2]  AEs related to IP= 1 (11.1%) [1]  Nausea= 1 (11.1%)  AEs related to study procedures= 1 (11.1%) [3]  **NNS-THS2.2:**  AEs= 2 (22.2%) [2]  SAEs= 0 [0]  Severe AEs= 0 [0]  AEs related to IP= 0 [0]  AEs related to study procedures= 0 [0] |
| NCT01967732 | Format: n (%) subjects; [n] AEs  **THS2.2-CC:**  AEs= 10 (45.5%) [21]  SAEs= 0  AE related to IP= 4 (18.2%) [4]  AE related to study procedure= 3 (13.6%) [7]  Mild AE= 7 (31.8%) [18]  Moderate AE= 3 (13.6%) [3]  Severe AE= 0  Discontinuation due to AE= 0  Dizziness= 5 (22.7%) [6]  Headache= 3 (13.6%) [3]  Presyncope= 1 (4.5%) [1]  Nausea= 2 (9.1%) [3]  Vomiting= 1 (4.5%) [1]  Muscle spasms= 1 (4.5%) [1]  Pallor= 1 (4.5%) [1]  Constipation= 1 (4.5%) [1]  Dysgeusia= 1 (4.5%) [1]  **CC-THS2.2:**  AEs= 7 (31.8%) [10]  SAEs= 0  AE related to IP= 4 (18.2%) [6]  AE related to study procedure= 0  Mild AE= 3 (13.6%) [5]  Moderate AE= 4 (18.2%) [5]  Severe AE= 0  Discontinuation due to AE= 0  Dizziness= 1 (4.5%) [1]  Headache= 2 (9.1%) [2]  Presyncope= 3 (13.6%) [3]  Nausea= 1 (4.5%) [1]  Vomiting= 2 (9.1%) [2]  Dysmenorrhoea= 1 (4.5%) [1]  **THS2.2-NNS:**  AEs= 4 (44.4%) [4]  SAEs= 0  AE related to IP= 3 (33.3%) [3]  AE related to NNS= 0  AE related to study procedure= 0  Mild AE= 2 (22.2%) [2]  Moderate AE= 2 (22.2%) [2]  Severe AE= 0  Discontinuation due to AE= 0  Headache= 1 (11.1%) [1]  Presyncope= 2 (22.2%) [2]  Pallor= 1 (11.1%) [1]  **NNS-THS2.2:**  AEs= 2 (22.2%) [4]  SAEs= 0  AE related to IP= 1 (11.1%) [1]  AE related to NNS= 0  AE related to study procedure= 0  Mild AE= 1 (11.1%) [3]  Moderate AE= 1 (11.1%) [1]  Severe AE= 0  Discontinuation due to AE= 0  Dizziness= 1 (11.1%) [1]  Muscle spasms= 1 (11.1%) [1]  Cough= 1 (11.1%) [1] |
| NCT01967706 | Format: n (%) subjects; [n] AEs  **THS-CC:**  AEs= 1 (4.5%) [1]  SAEs= 0 [0]  AEs related to IP= 0 [0]  AEs related to study procedure= 0 [0]  All AEs were mild.  **CC-THS:**  AEs= 2 (9.1%) [2]  SAEs= 0 [0]  AEs related to IP= 0 [0]  AEs related to study procedure= 0 [0]  All AEs were mild.  **THS-NRT:**  AEs= 0 [0]  SAEs= 0 [0]  AEs related to IP= 0 [0]  AEs related to study procedure= 0 [0]  All AEs were mild.  **NRT-THS:**  AEs= 1 (11.1%) [1]  SAEs= 0 [0]  AEs related to IP= 0 [0]  AEs related to study procedure= 0 [0]  All AEs were mild. |
| ISRCTN13439529 | 8 exposure period adverse events (AEs)[3 mild, 5 moderate] were reported by 6 of the 32 subjects (18.8%). One exposure period AE was related to cigarette use (cough with mild severity). The were no severe AEs.  6/23 participants (18.8%) reported 8 mild events (3 mild, 5 moderate). 1 events was related to CC use (cough with mild severity). There were no severe adverse events. |
| NCT01780688 | HTP exposure: 14 participants experienced AEs. Most frequent AEs: nausea (4 participants), headache (5), dizziness (4), presyncope (1).  CC exposure: 10 participants experienced AEs. Most frequent AEs: nausea (5 participants), headache (2), dizziness (2), presyncope (4), and abdominal pain (2).  Most AEs were mild. No AEs were reported by the investigator based on coughing symptoms. No notable changes in spirometry parameters were observed from baseline to the end of the study in either exposure group. |
| UMIN000017297 | HTP arm: 1 participant experienced an AE (vasovagal reaction) after PNTV product use.  CC arm: no AE was reported after CC1 smoking.  There were no serious AEs reported during the entire study. |
| NCT05114863 | "All product-related adverse events were categorised as mild in severity (e.g., dizziness, nausea and headache)" "No serious product-related adverse events observed following the use of PULZE 2.0 and iD/iSENZIA sticks" |
| NCT05459857 | "During the product trial period on Day −1, during which subjects were allowed to use a single iD of their choice with the Pulze HTS, one mild AE (dizziness) was reported by one (4%) subject. The Investigator considered this event unrelated to study product use. Overall, AEs were infrequently reported in this study, with six AEs reported by five (21%) subjects after study product randomisation. Catheter site pain was reported three times by three (13%) subjects, and the remaining AEs (constipation, dizziness, and neck pain) were reported by one (4%) subject each. The constipation and dizziness events were moderate in severity, and the catheter site pain and neck pain events were mild. The Investigator considered all AEs to be unlikely related or unrelated to study product." |
| NCT06093659 | "Overall, 15 AEs were reported, including eight AEs among six subjects (15.4%) in the non-menthol group and seven AEs among five subjects (13.9%) in the menthol group. |

# Supplementary Figure 6. Effect of heated tobacco product use compared with cigarette use (A), smoking abstinence (B) and e-cigarette use (C) on rate of participants reporting adverse events.


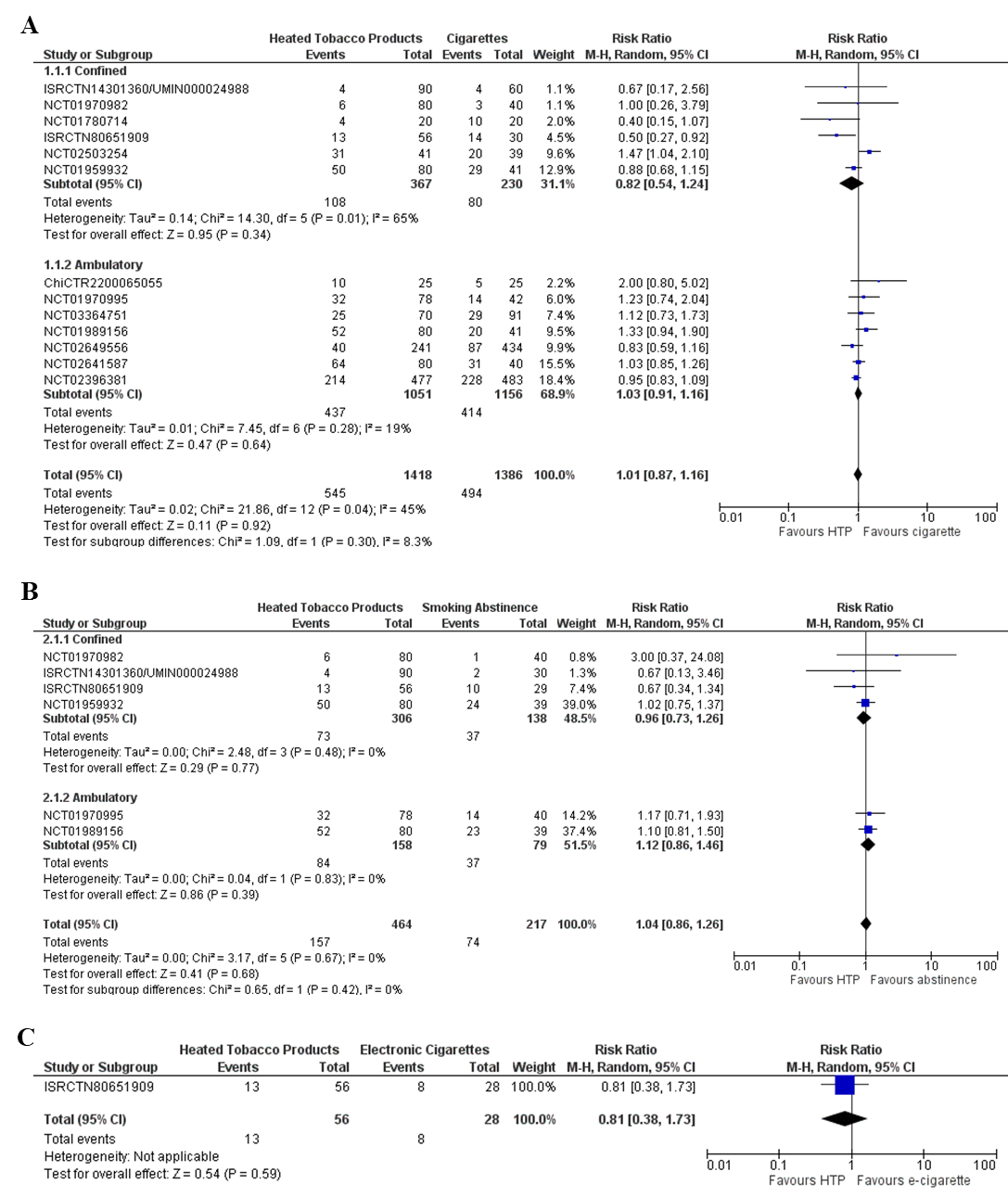


# Supplementary Figure 7. Effect of heated tobacco product use compared with cigarette use (A), smoking abstinence (B) and e-cigarette use (C) on rate of participants reporting serious adverse events.


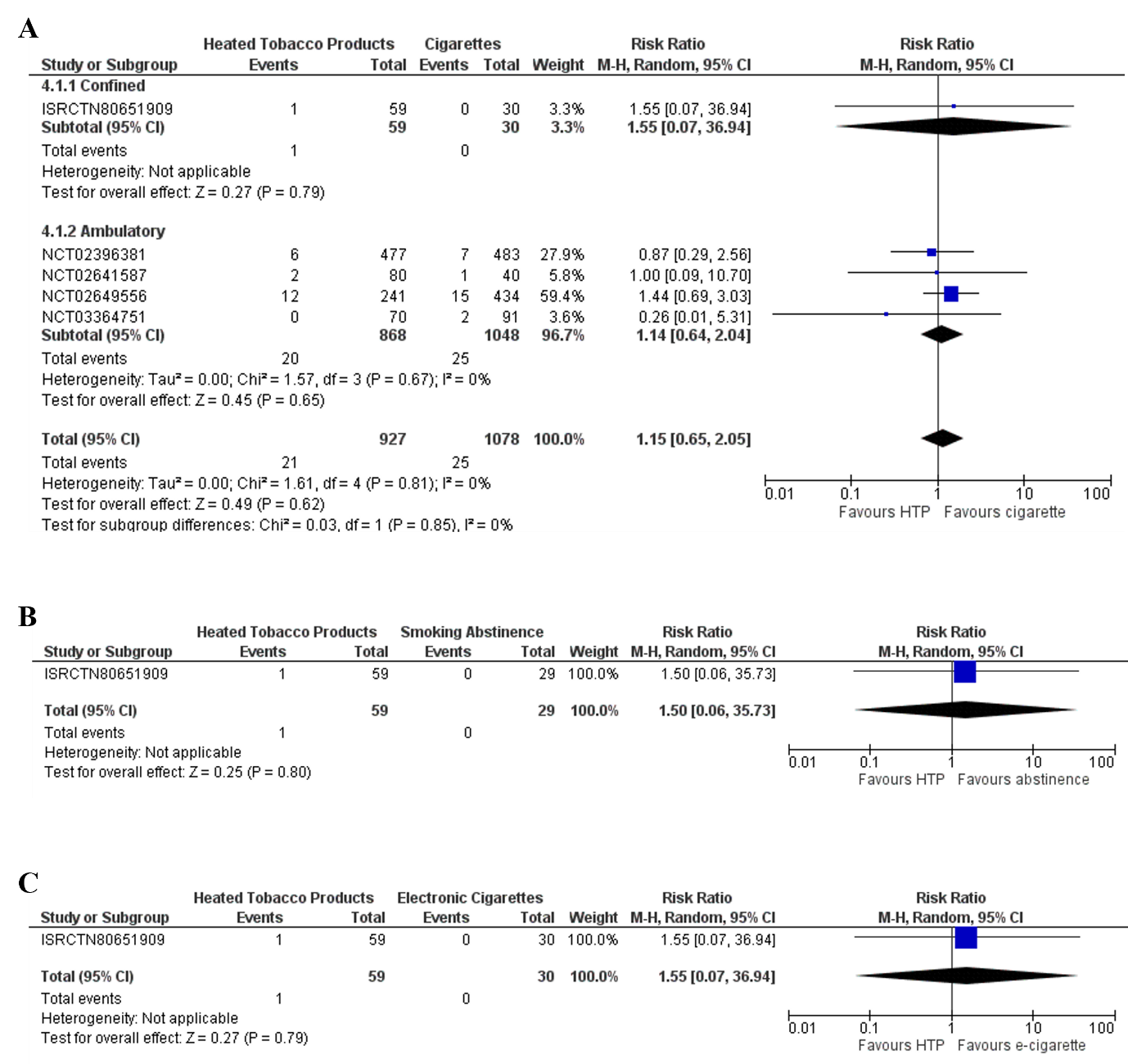

Supplement: online supplemental file 1 [file tc-35-3-s001.docx]
